# Improving the Performance of Carbon-Based Perovskite Solar Cells by the Incorporation of a Screen-Printed NiCo_2_O_4_ Interlayer

**DOI:** 10.1021/acsaem.4c01720

**Published:** 2025-01-16

**Authors:** Nidia G. García-Peña, Mahmoud Nabil, Dena Pourjafari, Diecenia Peralta-Domínguez, Wendy Yaznay Padrón-Hernández, Adriana P. Franco-Bacca, Araceli Ríos-Flores, Beatriz Eugenia Heredia-Cervera, Renan Escalante, Geonel Rodríguez Gattorno, Milenis Acosta, Paul Pistor, Juan Antonio Anta, Gerko Oskam

**Affiliations:** † Department of Applied Physics, CINVESTAV-IPN, Antigua Carretera a Progreso Km 6, Merida 97310, Yucatan, Mexico; ‡ Center for Nanoscience and Sustainable Technologies (CNATS), Department of Physical, Chemical and Natural Systems, 16772 Universidad Pablo de Olavide, Carretera de Utrera Km 1, 141013 Seville, Spain; § Facultad de Ingeniería Química, Universidad Autónoma de Yucatán, Periférico Norte, Kilómetro 33.5, Tablaje Catastral 13615, Chuburná de Hidalgo Inn., C.P. 97203 Merida Yucatan, Mexico; ∥ Facultad de Ingeniería, Universidad Autónoma de Yucatán, Avenida Industrias No Contaminantes por Anillo Periférico Norte, Merida 97203, Yucatan, Mexico

**Keywords:** nickel−cobaltite, coprecipitation, solar energy, perovskite photovoltaics, hole selectivity, pseudocapacitive oxide materials, electrochemical impedance spectroscopy

## Abstract

Hybrid lead halide perovskite solar cells (PSCs) stand out in terms of their high efficiency, yet the limited stability and process scalability pose challenges to their commercialization. Fully printable carbon-based perovskite solar cells (C-PSCs), consisting of a triple stack of mesoporous titania, zirconia, and carbon layers impregnated with the perovskite material, have been introduced as an attractive architecture; however, they generally exhibit lower efficiency. This study proposes a viable and scalable approach to increase the efficiency of C-PSCs by incorporation of an intermediate layer of mesoporous, nanostructured NiCo_2_O_4_ between the zirconia and carbon layers. The devices show an average increase in power conversion efficiency from 7.9 to 11%, with a champion device efficiency of 12.4%, associated with an enhanced average open-circuit voltage (*V*_OC_) from 0.869 to 0.962 V. Electrochemical impedance spectroscopy reveals that the high-frequency recombination resistance (*R*_HF_) decreases exponentially with *V*_OC_ with the same slope as for the reference triple-stack system, indicating that the mechanism is unchanged; however, a substantial increase in *R*_HF_ is observed. These results indicate that the hole extraction efficiency improves upon incorporation of the NiCo_2_O_4_ film thus decreasing surface recombination at the nonselective carbon contact. On the other hand, we postulate a possible contribution of the high capacitance of the interlayer, which may result in a shift of the Fermi energy of the carbon electrode and play a role in the hysteresis in the current - voltage curve.

## Graphical abstract

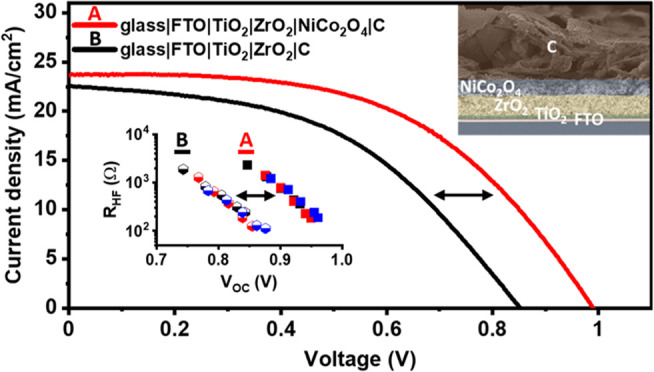

## 1 Introduction

Hybrid lead halide-based perovskite solar cells (PSCs) have emerged as a promising candidate for next-generation photovoltaics related to their use of abundant materials and low-cost fabrication processes, combined with high efficiencies evolving rapidly from 3.8% in 2009 ^1^ to the current efficiency record of 26.1%.^2^ Despite their potential, there is still a need for further improvements to make PSCs commercially viable, specifically in terms of stability, scalability, and materials costs.^3^ Over the past 10 years, a variety of architectures and configurations have been developed and implemented for scale-up and future commercialization, each with specific advantages and disadvantages. The first and most common architecture of high-efficiency PSCs is the n-i-p configuration, which usually employs mesoporous TiO_2_ as the electron transport layer (ETL), multiple cation and anion hybrid perovskites,^4,5^ organic materials, such as spiro-OMeTAD, as the hole transport layer (HTL),^6^ and noble metals such as gold or silver as the top contact.^7^ Solar cells are generally fabricated using spin coating, and this configuration has resulted in the highest efficiency; however, the materials and fabrication aspects increase production costs and limit scalability,^8^ while device stability is compromised due to a variety of related degradation mechanisms.^9–11^

More recently, p-i-n PSCs, where the HTL is deposited onto the transparent conducting oxide electrode, followed by the perovskite layer and finished by applying an ETL, have demonstrated significant progress in overcoming some of these challenges. Advances include the incorporation of more stable, inorganic HTLs such as based on NiO_*x*_,^12^ scalable deposition techniques such as slot-die coating thus improving production efficiency,^13^ and the replacement of noble metal contacts with cost-effective alternatives such as copper.^14^ This architecture often relies on fullerene-based materials, such as PCBM and C60, as ETLs, along with additional interface-modifying layers like BCP, which increase fabrication complexity and cost.^15^ To achieve optimal performance, NiO_*x*_ typically requires doping or specialized surface treatments to enhance its conductivity, modulate energy levels, and reduce defect densities.^16,17^ Additionally, the structural stability of the perovskite material can be affected by the passivation treatment of the NiO_*x*_ surface.^18^ Despite these limitations, p-i-n PSCs have currently reached high efficiencies similar to the n-i-p devices, and generally display better stability and scalability, making them strong contenders for commercialization.^19^

An alternative approach to overcoming the challenges in n-i-p PSCs was the introduction of the fully printable carbon-based perovskite solar cell (C-PSC).^20^ The architecture of C-PSCs is based on three mesoporous layers of TiO_2_, ZrO_2_, and carbon (C) that are screen-printed sequentially onto a transparent conductive substrate (glass/FTO). Afterward, the perovskite precursor is infiltrated into the mesoporous triple stack, crystallizing into the light-absorbing perovskite material upon annealing.^21^ The carbon-based material replaces the organic HTL and metal top contact in this structure, reducing production costs.^3^ Near-infrared processing can be used to decrease the heating time of mesoporous layers, resulting in greater cost savings.^22,23^ In addition, C-PSCs have shown superior long-term stability for both individual cells and modules.^24–26^ As an added production advantage, the full solar cell or module structure can be fabricated in ambient atmosphere, where only the infiltration process requires a better defined atmosphere, both for improved efficiency and process security related with the organic solvents used.^3,23^

Carbon as an inert and hydrophobic material has also been studied in n-i-p PSCs; however, the efficiency is generally lower than for systems that rely on an organic HTL and a metal top contact. In particular, implementation of a carbon hole extraction electrode generally results in a lower *V*_OC_, which has been attributed to the nonoptimal energy difference between the carbon Fermi level and the energy of the edge of the valence band (VB) of the perovskite and relatively inefficient hole transfer.^27,28^ Hence, to achieve high *V*_OC_ in carbon-based n-i-p PSCs it is essential to tune the Fermi energy of the carbon electrode and to improve the hole selectivity of the perovskite/carbon interface.

Similar observations have been reported for the printed triple stack C-PSCs, and several methods have been explored to optimize hole collection, including the integration of printable p-type inorganic layers such as NiO^29,30^ and Co_2_O_3_.^31^ However, the precise reasons for the improvement are still unclear. Recently, impressive record efficiencies of 19.74 ^32^ and 22.2%^33^ were reported by the group of Hongwei Han, where they use a highly optimized, modified carbon electrode in combination with interface treatments to maximize the charge collection efficiency and reduce recombination at the TiO_2_/perovskite interface to achieve record performance.

In this work, we have integrated a screen-printed mesoporous layer of NiCo_2_O_4_ into the typical C-PSC structure between the ZrO_2_ and C layers and focus on the use of electrochemical impedance spectroscopy (EIS) to elucidate the mechanisms through which the presence of the interlayer may affect the solar cell performance. NiCo_2_O_4_ is a low-cost, mixed-valence material and has been reported to function as a p-type semiconductor^34^ with more electrochemical active sites and higher conductivity than NiO or Co_3_O_4_.^35^ Additionally, it has a high theoretical specific capacitance^36^ and both reductive and oxidative electrocatalytic properties.^37^ NiCo_2_O_4_ can be synthesized and deposited using various physical and chemical methods, including RF-sputtering,^38^ pulsed laser deposition,^39^ solvothermal,^40^ template-assisted,^41^ coprecipitation,^42^ and sol–gel^43^ as well as atomic layer deposition,^44^ electrodeposition,^45^ spray pyrolysis,^46^ chemical bath deposition,^47^ spin coating,^48^ and inkjet printing.^37^ NiCo_2_O_4_, owing to its versatile characteristics, has recently found its application in a wide array of fields. In addition, it has been used as an electrocatalyst, for instance, in the electrolysis of water waste^49^ and water splitting for clean energy production.^50^ It is also widely used as an electrode material for supercapacitors, either alone,^51,52^ or combined with carbon.^53,54^ It has been applied in developing gas sensors^55^ and as an anode material for lithium-ion batteries.^56^ In the field of solar cells, NiCo_2_O_4_ has been used as a counter electrode in dye-sensitized solar cells (DSSCs) alone^57^ and as a composite with carbon.^58^ Interestingly, it has also been reported as a photocathode in p-type DSSCs^59^ and as HTL in p-i-n PSCs.^60,61^

From the nanostructured NiCo_2_O_4_ powder we formulated a paste with the proper rheological properties for successful film deposition using screen printing. We fabricated C-PSCs with the newly integrated layer (glass|FTO|TiO_2_|ZrO_2_|NiCo_2_O_4_|C), finding a significant improvement in solar cell performance compared to that of the traditional triple-stack configuration (glass|FTO|TiO_2_|ZrO_2_|C). We use electrochemical impedance spectroscopy (EIS) to elucidate the mechanisms that lead to this performance enhancement in the devices with the incorporated NiCo_2_O_4_ interlayer.

## 2 Results and Discussion

### 2.1 Synthesis and Characterization of NiCo_2_O_4_

NiCo_2_O_4_ was synthesized through a green, aqueous coprecipitation method using the respective nitrate salts with NaOH/H_2_O_2_ under ambient pressure and temperature. Based on thermogravimetric analysis (TGA; Figure S1), the sintering step at 400 °C results in the dehydrated oxide as a black powder. The purity and crystallinity of the product were confirmed with X-ray diffraction (Figure S2). This was supported by the Raman spectrum (Figure S3), which shows 5 signals in the 100 to 1000 cm^–1^ range, corresponding to the F_2g_, E_g_, F_2g_, F_2g_, and A_1g_ Raman-active modes from MCo_2_O_4_ spinel structures.^62,63^ A detailed XPS study was undertaken (Figure S4) showing the high degree of formation of the NiCo_2_O_4_ spinel structure; it is worth noting that some Co^2+^ may persist despite H_2_O_2_ oxidation. The results indicate the possible presence of Co^3+^ in the tetrahedral site,^64^ which exists in the NiCo_2_O_4_ oxide, while separate peak arises from oxygen attached to Co^3+^ in the octahedral coordination.^65^ Refer to the Supporting Information for a detailed discussion of the XPS results. UV–vis diffuse reflectance spectroscopy (Figure S5) identifies two direct band gaps at 1.98 and 2.53 eV. The first corresponds to the usual reported value for this compound,^66^ while the latter was somewhat unexpected but consistent with previous studies on NiCo_2_O_4_ hexagonal nanoplatelets.^67^ The coexistence of Co^2+^ and Co^3+^ in high-spin and low-spin states has been suggested as the reason for the two band gaps^68^ with the observation of Co^3+^ both in tetrahedral (high-spin) and octahedral (low-spin) coordination sites. The presence of small amounts of Co^2+^ incorporated into tetrahedral sites was also suggested as a possible explanation for the observed results.

Scanning electron microscopy (SEM) was used to analyze the black powder, confirming the presence of hexagonal nanoplatelets. The micrographs in Figure S6a,b show nanoplatelets with a core-ring structure, consistent with the observation of two direct band gaps.^67^ The crystals have an average diameter of 200 nm, ranging from 80 to 320 nm, and a thickness of approximately 50 nm. The size histogram can be found in the inset of Figure S6a. The NiCo_2_O_4_ powder was processed to produce a suitable paste for the screen-printing technique.

Figure 1a shows SEM images of the screen-printed layer at different magnifications. The deposited layer is smooth and uniform without particle agglomeration. Particles retain their original crystallite size and form despite undergoing the milling process. As expected, the deposited layer is mesoporous, a fundamental property that allows for the infiltration of the perovskite precursor solution. Contact angle analysis was performed on a deposited and annealed sample to ensure that the perovskite precursor would correctly infiltrate the NiCo_2_O_4_ layer. Figure 1b shows the angle of the perovskite precursor solution droplet on the surface of the printed layer. A small contact angle of 18° indicates excellent wettability. The experiment was repeated five times to ensure the test reliability, obtaining approximately the same result each time. XRD, Raman, and XPS analyses were conducted on the printed layer confirming that the paste fabrication and screen-printing process did not alter the material properties.

**Figure 1:**
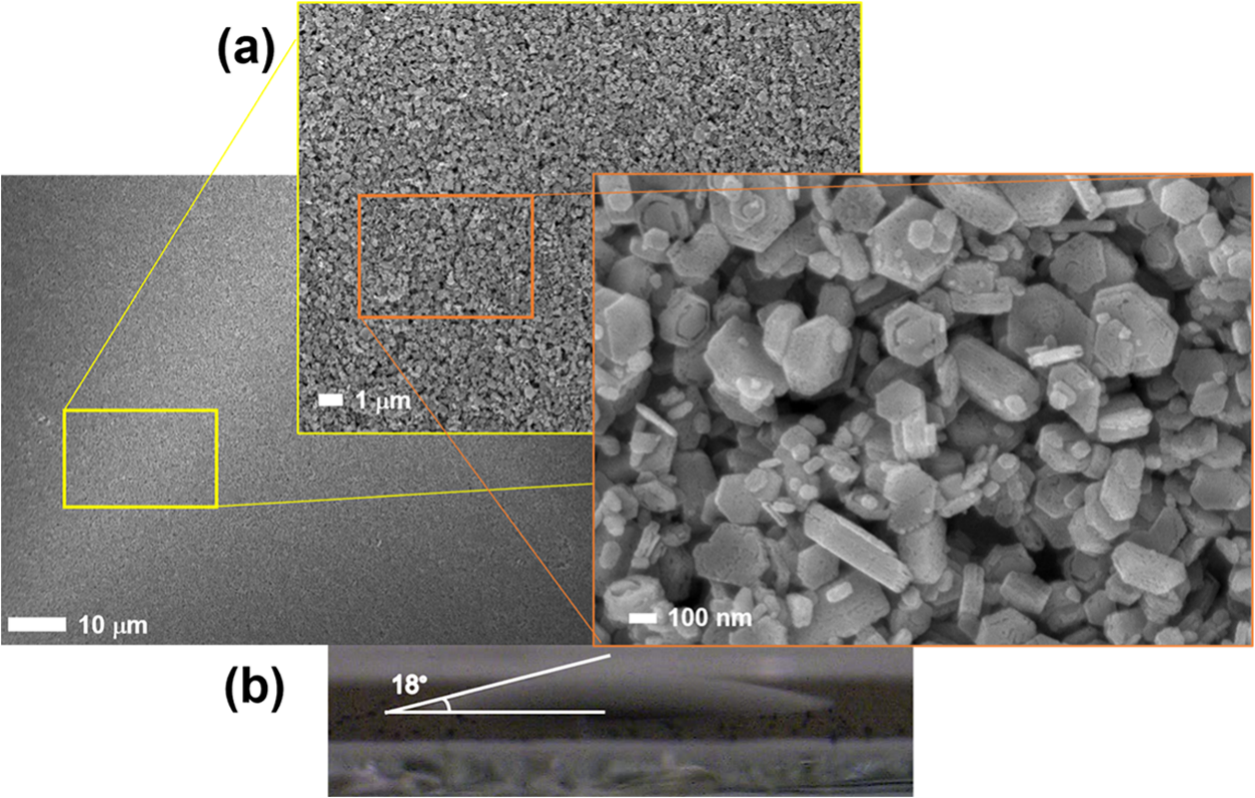
(a) SEM images of the screen-printed NiCo_2_O_4_ layer at different magnifications. (b) Contact angle of a perovskite precursor solution drop deposited onto a screen-printed NiCo_2_O_4_ layer.

### 2.2 Influence of NiCo_2_O_4_ Interlayer on the Performance of C-PSCs

Solar cells were fabricated both in the conventional triple-stack (glass|FTO|TiO_2_|ZrO_2_|C) architecture and in the new configuration with the NiCo_2_O_4_ layer in between the ZrO_2_ and C layers (glass|FTO|TiO_2_|ZrO_2_|NiCo_2_O_4_|C). The full device configuration is illustrated in Figure S8. Figure 2 shows the resulting mesoporous structure. The inclusion of the new interlayer did not affect the screen-printing of the carbon film. The thickness of each coating was determined using cross-sectional SEM micrographs. The TiO_2_, ZrO_2_, and C layers measured approximately 600 nm, 2.7 μm, and 14 μm, respectively. To investigate the impact of the NiCo_2_O_4_ interlayer thickness, two sets of cells were prepared. The first was deposited using the original, optimized NiCo_2_O_4_ paste formulation, resulting in a 2.1 μm layer with excellent morphology and uniformity, as evident in the cross-sectional SEM images (Figure 2a). A thinner, 1.1 μm layer was deposited by diluting the original paste with α-terpineol at a 1:1 ratio. However, the 1.1 μm layer exhibited reduced uniformity, as seen in Figure 2b, which may be due to the suboptimal rheological properties introduced by dilution; nonetheless, no pinholes were detected. This inhomogeneity likely contributed to nonideal infiltration of the perovskite precursor, which may compromise the charge extraction efficiency and negatively impact the solar cell performance.

**Figure 2:**
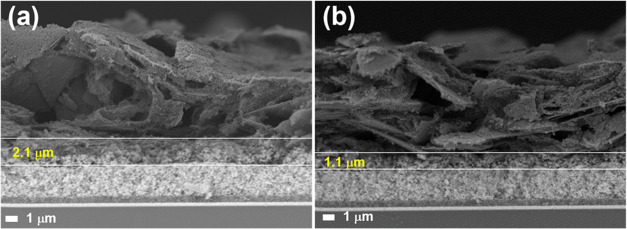
Screen-printed mesoporous stacks with an integrated NiCo_2_O_4_ layer of two different thicknesses: (a) 2.1 μm from original paste; and (b) 1.1 μm from diluted paste.

SEM analysis was performed on cells with the two different NiCo_2_O_4_ thicknesses before and after perovskite infiltration. The images were captured under the LABE (low angle backscattered electrons) scanning mode, where brighter zones indicate atoms with higher atomic numbers, such as lead. As shown in Figure S7, the perovskite crystals were fully embedded in the mesoporous TiO_2_, ZrO_2_, and NiCo_2_O_4_ layers in both types of stack. The extent of infiltration was so high that it was difficult to distinguish between the different oxide coatings. Perovskite crystals were also found in the carbon layer; these results are consistent with correctly infiltrated C-PSCs.

Figure 3 shows the current density*–*voltage curves (*J–V* curves) and photovoltaic parameters of the best-performing cell from each configuration, i.e., C-PSCs with a 2.1 μm thick NiCo_2_O_4_ layer, a 1.1 μm thick NiCo_2_O_4_ layer, and the triple-stack reference devices. The solar cells with the 2.1 μm thick NiCo_2_O_4_ interlayer achieved a champion power conversion efficiency (PCE) of 12.4%, compared to 9.1 and 9.7% for the reference solar cells and the devices with the 1.1 μm thick NiCo_2_O_4_ interlayers, respectively. Incorporating the NiCo_2_O_4_ layer results in an increased PCE through improvement of all the photovoltaic parameters, including short-circuit current density (*J*_SC_) and fill factor (FF), but most importantly by a significant increase of the *V*_OC_. The *V*_OC_ increased to 0.991 V for the device with the 2.1 μm thick NiCo_2_O_4_ interlayer compared to 0.854 V for the reference device. The efficiencies obtained in this work are lower than the reported records for screen-printed C-PSCs; however, our devices demonstrate competitive *V*_OC_ and *J*_SC_ values, which are in line with the best-performing cells reported in the field (see, for example, ref 65). As shown in Tables S1–S3, the primary limitation in device efficiency stems from the low fill factor (FF) of around 0.4–0.5. The lower FF values are attributed to the carbon paste used in the fabrication process and are related to the series resistance of the solar cell (see Section 2.3); this represents a practical issue that does not negate the fundamental performance improvement through the increase of *V*_OC_. Figure 4 shows the box charts of the photovoltaic parameters of 10 solar cells for each configuration; the details are provided in the Supporting Information (Tables S1–S3). Figure S9 in the Supporting Information shows the corresponding current–voltage curves measured at 50 mV s^–1^.

**Figure 3:**
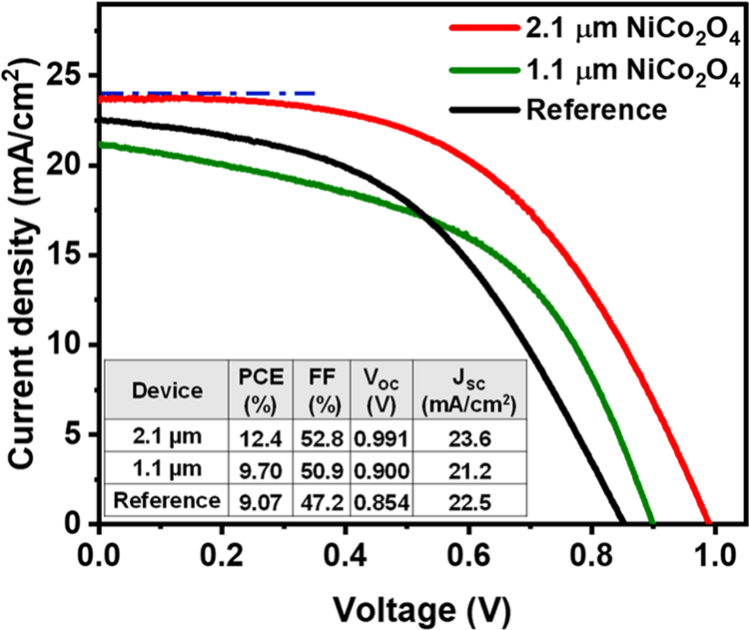
Current density–voltage curves (reverse scan) of the champion cells of each type (1.1 μm thick NiCo_2_O_4_ layer, 2.1 μm thick NiCo_2_O_4_ layer, and reference), along with their photovoltaic parameters.

**Figure 4:**
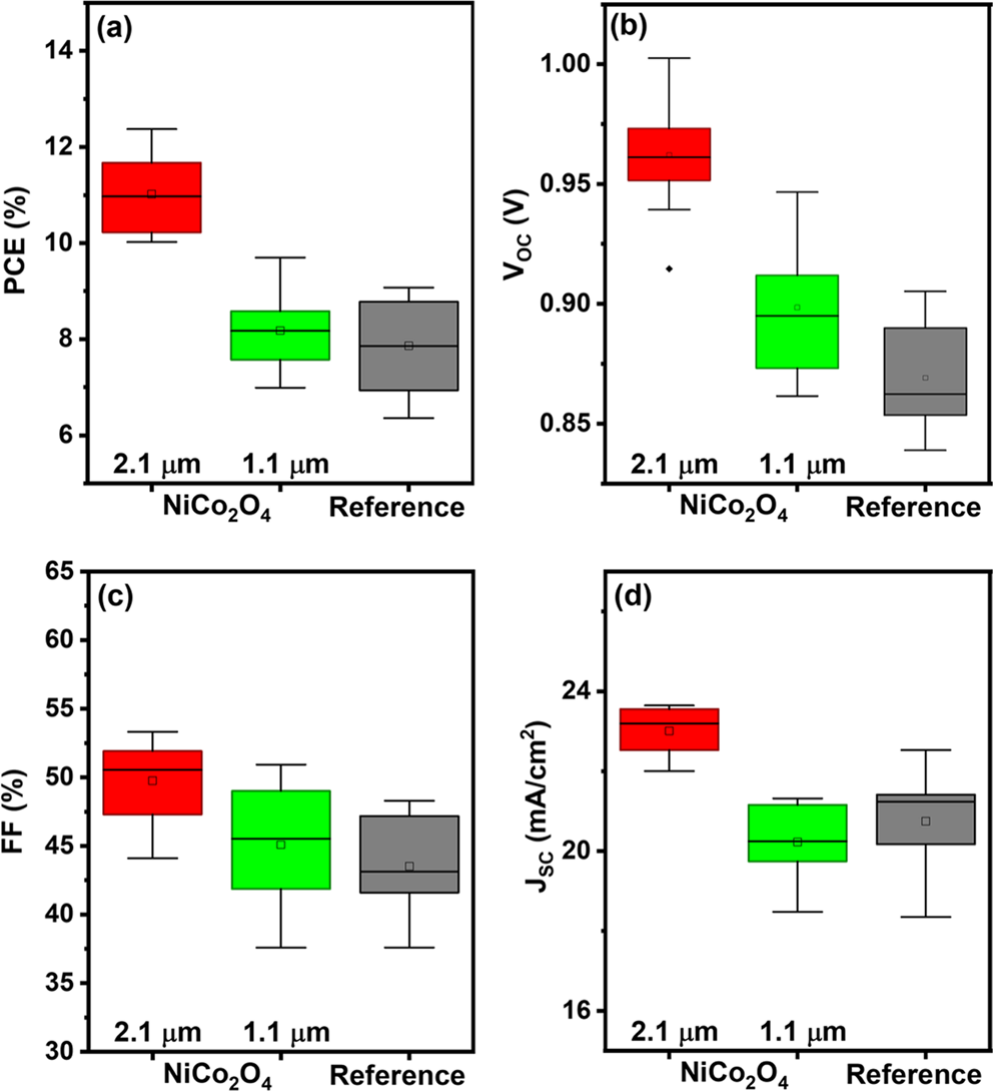
Photovoltaic parameters statistics acquired from 10 devices for each solar cell configuration. The data were recorded from the reverse scans: (a) power conversion efficiency; (b) open-circuit voltage; (c) fill factor; and (d) short-circuit photocurrent density. The boxes depict the interquartile range (IQR), with the median marked by the line dividing the boxes. The whiskers extend to the furthest data points within 1.5 times the IQR from the top and bottom of the box. Mean values are indicated by open squares, while outlier data points are represented by black diamonds.

As can be observed in Figure S9, the hysteresis increases with the thickness of the NiCo_2_O_4_ interlayer. The hysteresis of PSCs has been well-documented both in the n-i-p and p-i-n configurations, and this phenomenon is generally attributed to ion movement; in addition, a large hysteresis is generally considered a threat to stability.^69–72^ The hysteresis has been observed to increase with decreasing grain size, and the associated slowing down of ion movement is reflected by a concomitant decrease in the frequency of the resulting signal in the optical and impedance spectra.^61–63^ In the mesoporous C-PSC, the dominant grain size of the perovskite is smaller than 50 nm; hence, ion movement is expected to be very slow. In addition, it was recently shown that irreversible ion movement effects may result in a “bump” in the *J–V* curve that strongly depends on the scan rate and which may result in a lower steady-state efficiency.^73^ On the other hand, the dependence of the hysteresis on the NiCo_2_O_4_ interlayer thickness in our cells implies that besides ion movement, a charging effect related to the high pseudocapacitance of the material may also play a role. In this case, the high pseudocapacitance of the material and the corresponding reversible charging may also be beneficial and partially responsible for the increase in the *V*_OC_ of the devices. In this scenario, the larger hysteresis is not necessarily related to lower stability.

The average PCE value of solar cells with the 2.1 μm NiCo_2_O_4_ interlayer was 11.0%, a significant improvement compared to the average PCE of the reference cells of 7.86%. For the solar cells with the 1.1 μm NiCo_2_O_4_ interlayer, the average PCE is also slightly larger at 8.18% due to an increase of *V*_OC_ and FF, although the average *J*_SC_ decreases somewhat. The reduction in *J*_SC_ for the 1.1 μm layer can be attributed to its nonuniform morphology, as discussed in Figure 2 and Section 2.2, which likely impeded the perovskite precursor from infiltrating properly, hindering efficient charge extraction. Thus, optimizing the paste formulation for each thickness is critical to maintain layer uniformity and ensure consistent device performance. Further studies are under way to determine the optimal thickness of the NiCo_2_O_4_ interlayer. In general, however, it can be concluded that the incorporation of the NiCo_2_O_4_ interlayer improves the C-PSC performance.

### 2.3 Influence of NiCo_2_O_4_ Interlayer: Electrical Impedance Spectroscopy (EIS)

In order to get insight on how the presence of the NiCo_2_O_4_ interlayer contributes to the performance of C-PSCs, EIS measurements were carried out at *V*_OC_, where recombination equals generation, as a function of the light intensity. We used three LED light sources of different wavelengths, including white, blue (480 nm), and red (650 nm). Figure 5 displays Nyquist plots corresponding to measurements under blue illumination at varying irradiance levels for standard triple-stack reference cells and cells with the 2.1 μm NiCo_2_O_4_ interlayer.

**Figure 5:**
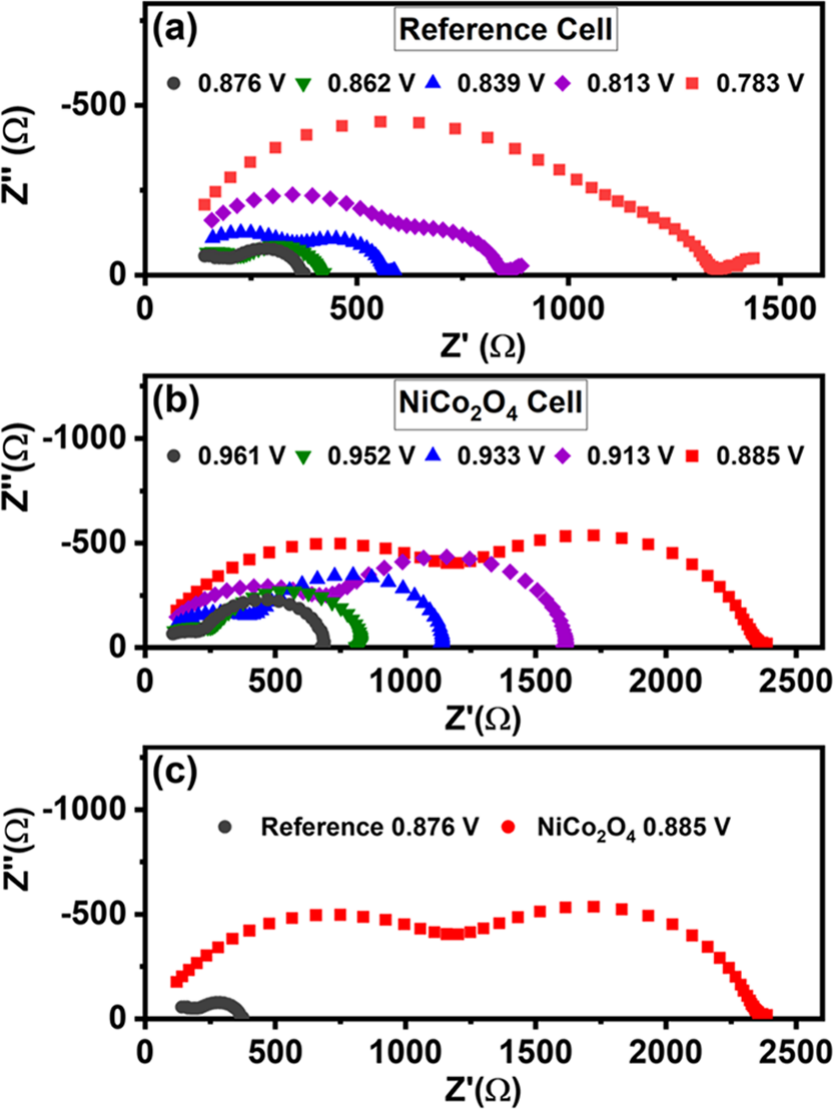
Nyquist plots obtained by impedance spectroscopy for: (a) cells with the 2.1 μm NiCo_2_O_4_ interlayer; and (b) standard triple-stack reference cells. Figure (c) shows a comparison between the spectra obtained for the two cell types at similar *V*_OC_.

For both cell configurations, two main features dominate the spectra. For the reference cells, the two semicircles tend to merge at lower *V*_OC_ values, while this is not observed for the cells with the interlayer. Apart from the two semicircles, a lower frequency signal is detected in the reference cells but not in the cells with NiCo_2_O_4_. The Cole–Cole plots illustrate that the apex of both semicircles, describing the respective time constants, for both types of solar cell shift to higher frequencies as the light intensity and *V*_OC_ increase (see Figure S10 in the Supporting Information). Figure 5c illustrates the significant difference in the impedance spectra of both cell types at similar *V*_OC_.

The impedance spectra were analyzed using a standard equivalent circuit of a resistance (*R*_S_) in series with two parallel RC elements, describing the two time constants observed at high frequency (HF) and low frequency (LF).^74^ The equivalent circuit is illustrated in Figure S11­(a). The series resistance of the solar cells is relatively high in both configurations (reference and NiCo_2_O_4_-integrated cells), as demonstrated in Figure S11­(b). The *R*_S_ values extracted from the EIS spectra are independent of *V*_OC_ as expected. The relatively high values for *R*_S_ are consistent with the relatively low fill factor (FF) of our cells, as compared to the cells with record efficiencies reported in the literature. We attribute the high series resistance to the carbon layer as well as the overall architecture of the device, both of which are under further optimization. As shown in Figure S11­(b), *R*_S_ decreases upon the incorporation of the NiCo_2_O_4_ layer, which is an interesting observation given the additional oxide layer. This can be attributed to the enhanced selectivity and improved charge transport properties facilitated by the NiCo_2_O_4_ layer, reducing resistive losses at the back contact. The decrease of R_S_ also correlates with the larger FF obtained for the solar cells with the NiCo_2_O_4_ interlayer.

Figure 6 displays the resistive (*R*_HF_ and *R*_LF_), capacitive (*C*_HF_ and *C*_LF_), and time constants (τ_HF_ and τ_LF_) as a function of the *V*_OC_ generated using different light intensities and the three LEDs. In both the reference cells and cells with the NiCo_2_O_4_ interlayer, the values for *R*_HF_ and *R*_LF_ decrease exponentially as the *V*_OC_ increases, which is typical behavior of a recombination resistance.^75,76^ The recombination resistance for both the HF and LF signals increases significantly when the NiCo_2_O_4_ interlayer is incorporated; this is evident when the Nyquist spectra are compared at similar *V*_OC_, as shown in Figures 5c and 6a,b.

**Figure 6:**
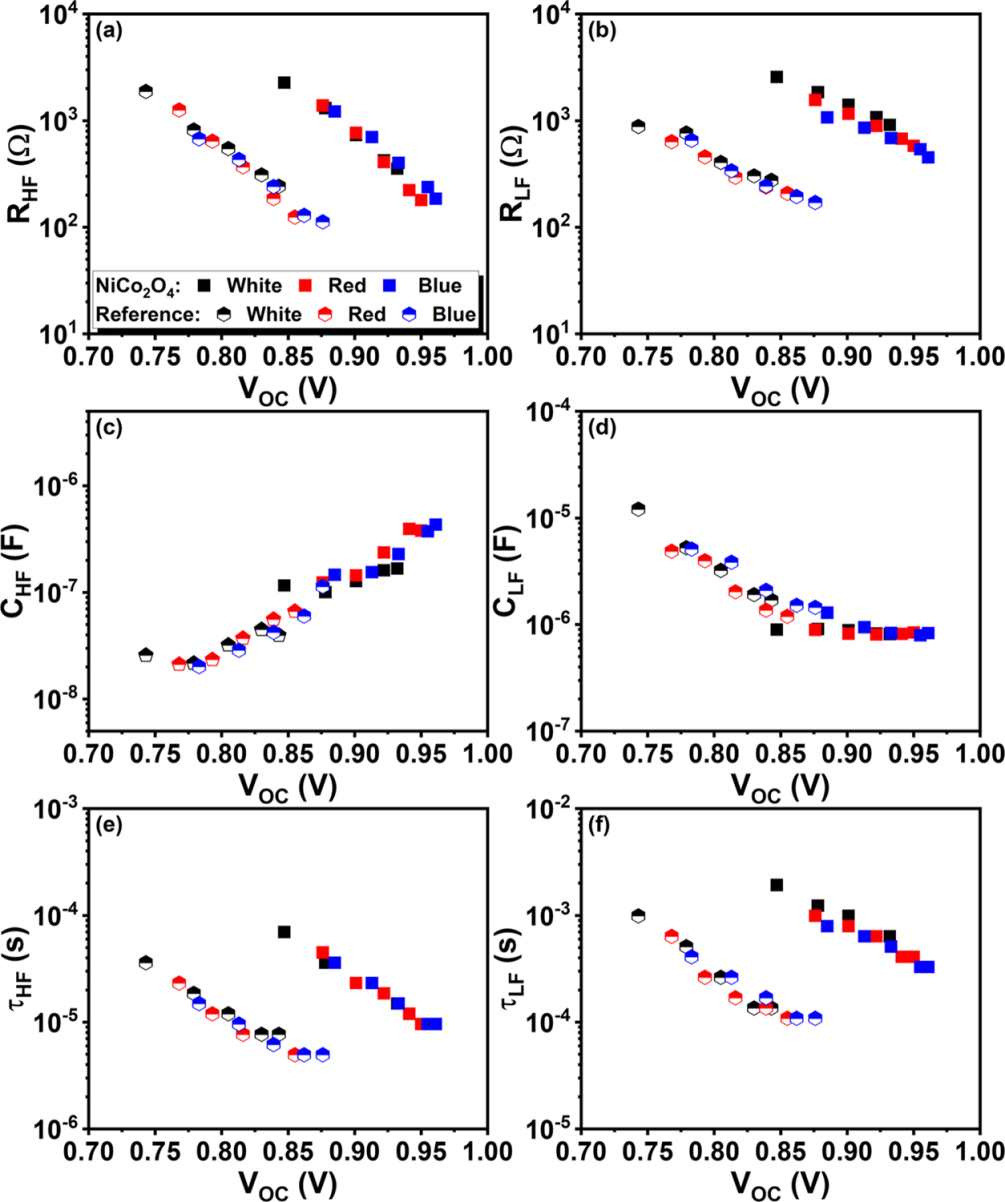
Dependence of (a, b) the resistive elements (*R*_HF_ and *R*_LF_); (c, d) the capacitive elements (*C*_HF_ and *C*_LF_); and (e, f) the time constants (τ_HF_ and τ_LF_), as a function of *V*_OC_ (generated by modifying the intensity of the 3 different LEDs) for reference and NiCo_2_O_4_ devices.

The low-frequency capacitance shows a more complex dependence with voltage: *C*_LF_ of the reference cells first decreases with increasing *V*_OC_ at lower voltages, and stabilizes at higher *V*_OC_. For the cells with the NiCo_2_O_4_ interlayer, *C*_LF_ has a similar value as for the reference cells in the range of 0.85–0.88 V and remains independent upon further increase of *V*_OC_. The interpretation of the low frequency results is complicated: it appears that the resistance (Figure 6b) is determined by recombination but with a smaller slope than that of the high-frequency resistance (Figure 6a). The low-frequency capacitance is often found to increase with *V*_OC_, while in this case, the trend is partially opposite. However, the here-defined low-frequency signal does not appear to correspond to the typical low-frequency signal related to ion movement processes, but rather to what is usually called the medium-frequency signal in conventional PSCs.^77^

For both configurations, the time constants τ_HF_ and τ_LF_ decrease exponentially with increasing *V*_OC_, which is commonly found for conventional PSCs for the high and medium-frequency signals. The time constant with strong ionic influence observed in conventional PSCs is generally independent of *V*_OC_, reinforcing the suggestion that ionic movement is not observed in the usual measurement range.

The ideality factor of the solar cells with and without NiCo_2_O_4_ interlayer can be calculated from the slope of the ln­(*R*_HF_) vs *V*_OC_/*k*_B_ *T* plot, where *k*_B_ is the Boltzmann constant and *T* is the absolute temperature.^78,79^ The HF signal showed typical ideality factor values, varying between 1.4 and 1.9. Values close to 1 are generally associated with surface recombination, while a value closer to 2 is observed when Shockley-Read-Hall (SRH) recombination in the bulk dominates.^80–82^ The values are in between indicating a mix of both processes; there is no significant difference in the ideality factors calculated for cells with and without a NiCo_2_O_4_ layer, suggesting similar recombination mechanisms for the two cell configurations.

We can observe from Figure 6a that the additional NiCo_2_O_4_ layer significantly increases the recombination resistance, which correlates with an increase of the *V*_OC_. Kerremans et al. has demonstrated the direct effect of contact selectivity on the *V*_OC_,^83^ and the results indicate that the NiCo_2_O_4_ interlayer increases the hole selectivity at the carbon contact, which may be related to the reported p-type conductivity of NiCo_2_O_4_.^34,60,61^ This improved selectivity facilitates the hole extraction step and prevents electron extraction at the carbon interface, thus reducing recombination at a nonselective carbon contact. This interpretation has been reported previously for other systems,^31,84^ and is in agreement with the significant increase observed for *R*_HF_ and *R*_LF_. As shown by Kerremans et al., light absorption occurs mainly by the perovskite in the mesoporous TiO_2_ layer, even under red LED illumination^83^ As a consequence, upon increasing the thickness of the layers and increasing the distance that electrons would need to travel to recombine at the nonselective interface, this effect becomes less relevant.^33,83^ It was recently demonstrated using a combination of theoretical and experimental studies, that the recombination resistance is correlated with degradation and tends to decrease with time.^85^ The significant increase in recombination resistance upon incorporation of the NiCo_2_O_4_ interlayer, reflected in both *R*_*HF*_ and *R*_*LF*_, may also indicate improved solar cell stability. We are currently pursuing prolonged stability measurements for both cells and mini modules to test this expectation.

The C-PSC cells in this work are characterized by a significant layer thickness, with the TiO_2_ film thickness of 0.6 μm and the ZrO_2_ film thickness of 2.7 μm, and this further increases with the 2.1 μm thick NiCo_2_O_4_ layer; hence, this increase in layer thickness could result in better selectivity. It should be noted that the carbon film thickness is very large, but this is mainly to minimize serial resistance. On the other hand, Figure 6 shows that the results are essentially independent of the color of the illumination, i.e., the difference in absorption depth of red and blue light does not lead to a change in collection efficiency. These results indicate that the diffusion length of the carriers is sufficient for efficient collection, but also to permit surface recombination at the nonselective carbon contact.

Keeping in mind the already large active layer thickness, another explanation for the increase of *V*_OC_ may be related to the high specific capacitance of NiCo_2_O_4_.^36^ Numerous applications of this oxide material make use of this characteristic property, for example, as an electrode in supercapacitors^51,52^ and solar cells,^57,59^ by enhancing the specific capacitance of carbon and improving its electrode function.^53,54,58^

A recent study has shown that introducing Mn_3_P_2_O_8_ into graphene increases its specific capacitance, leading to a shift of the Fermi level.^86^ We postulate that a similar effect may occur in C-PSCs with the NiCo_2_O_4_ interlayer: an indirect indication is provided by the hysteresis in the *J–V* curves, which increases proportionally with the NiCo_2_O_4_ layer thickness. The hysteresis is most likely linked to the capacitive behavior of the NiCo_2_O_4_ layer, rather than only to ionic movement, in concordance with the EIS results. The presence of NiCo_2_O_4_ increases the specific capacitance of the electrode and a voltage drop over this layer may result in a shift of the Fermi level of the carbon electrode to lower energy, thus decreasing the energy offset between the electrode Fermi level and the perovskite VB level, as illustrated in Figure 7, and increase the *V*_OC_. Note that at this stage there is no direct evidence for this possible effect. In summary, the substantial increase in *V*_OC_ upon incorporating layers with high specific capacitance between the ZrO_2_ and carbon layer in the C-PSC structure may be due to a combination of improved hole selectivity of the carbon contact and a downshift of the carbon Fermi level with respect to the situation where the interlayer is not present.

**Figure 7:**
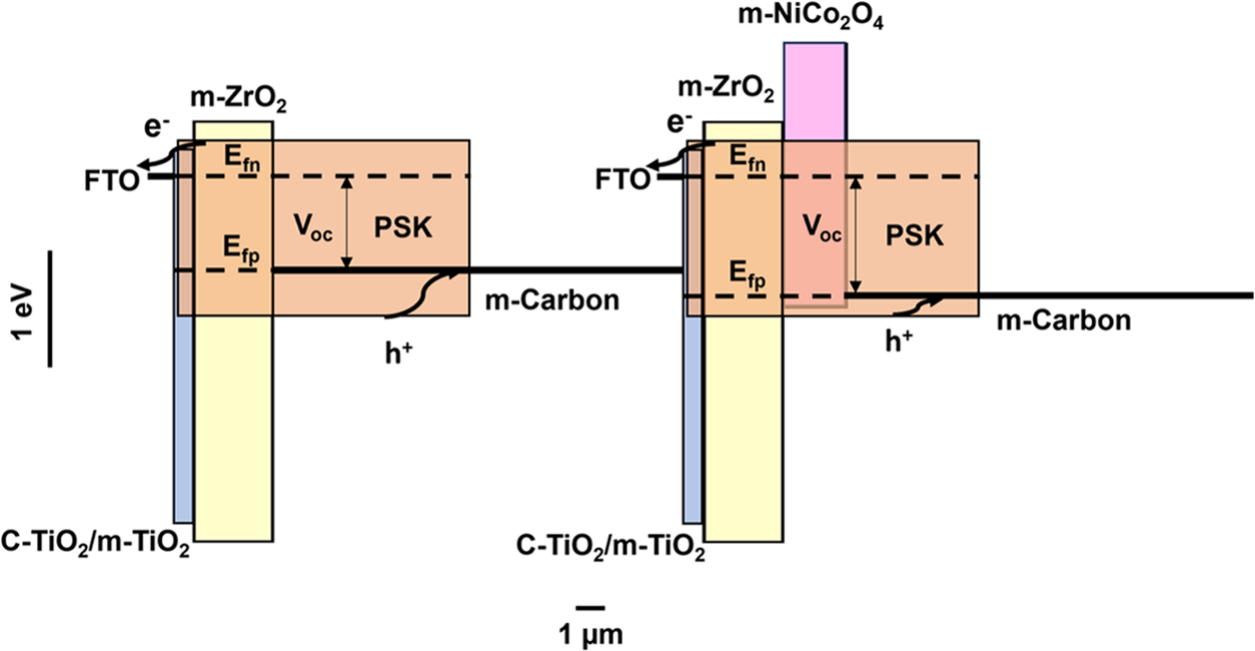
Schematic diagram illustrating the relative thicknesses of the TiO_2_, ZrO_2_, and NiCo_2_O_4_ layers, where PSK is short for perovskite; the effect of the incorporated NiCo_2_O_4_ layer on the hole extraction dynamics is highlighted.

## 3 Conclusions

Nanostructured NiCo_2_O_4_ has been synthesized using a coprecipitation method followed by an optimized annealing process to obtain the adequate properties to be used as an additional functional interlayer in the fully printed C-PSC. A 2.1 μm thick NiCo_2_O_4_ layer exhibiting excellent uniformity, porosity, and wettability was integrated using the screen-printing technique between the ZrO_2_ and C printed layers. The incorporation of the NiCo_2_O_4_ interlayer leads to a significant improvement in the efficiency of C-PSCs. The champion device exhibited a remarkable PCE of 12.4%, significantly outperforming the best-performing reference device. This improvement in PCE is due to increased values for the *J*_SC_, FF, and, in particular, *V*_OC_. EIS measurements show that incorporating the NiCo_2_O_4_ layer significantly increases the recombination resistance, which can be related to improved hole selectivity of the carbon contact. Additionally, we propose that a downshift of the carbon electrode Fermi level as a result of the capacitive effect of NiCo_2_O_4_ may be partially responsible for the increase in *V*_OC_.

## 4 Experimental Methods

### 4.1 Materials

For the synthesis and paste fabrication the following reagents were used: Co­(NO_3_)_2_·6H_2_O (98% reagent grade), Ni­(NO_3_)_2_·6H_2_O (98% reagent grade), NaOH (≥97% ACS reagent pellets), H_2_O_2_ (30 wt %), ethyl cellulose (viscosity 100 cP, 5% in toluene/ethanol 80:20), terpineol (anhydrous mixture of isomers), ethanol (≥99.5% ACS reagent), titanium diisopropoxide bis­(acetylacetonate) (75 wt % in isopropanol), 2-propanol (anhydrous, 99.5%), lead­(II) iodide (98% powder), methanol (anhydrous, 99.8%), γ-valerolactone (≥99%, ReagentPlus), and α-terpineol (90%, technical grade). All reagents were purchased from Sigma-Aldrich, except for methylammonium iodide (MAI, >99.99%) and 5-ammonium valeric acid iodide (5-AVAI, >99%), which were obtained from GreatCell Solar Materials (Australia). All reagents were used as received without further purification.

For the C-PSC devices fabrication, mesoporous TiO_2_ (30 NR-D) paste was purchased from GreatCell Solar Materials (Australia), mesoporous ZrO_2_ (Zr-Nanoxide ZT/SP) was acquired from Solaronix (Switzerland), and conductor carbon paste was obtained from GEM (United Kingdom).

### 4.2 Synthesis of NiCo_2_O_4_

NiCo_2_O_4_ oxide was synthesized through a simple coprecipitation method. In brief, 8.731 g (30 mmol) Co­(NO_3_)_2_·6H_2_O and 4.362 g (155 mmol) Ni­(NO_3_)_2_·6H_2_O were dissolved in 150 mL deionized (DI) water. Then, 10 g (250 mmol) NaOH, previously dissolved in 50 mL DI water, was added dropwise with magnetic agitation. The mixture was maintained with stirring for 1 h, and afterward 40 mL concentrated H_2_O_2_ (30 wt %) was slowly added. The dark-colored suspension was kept under magnetic agitation overnight. The next day, the mixture was centrifuged at 8000 rpm for 10 min. The brown solid was washed with 3 × 50 mL DI water, 3 × 50 mL ethanol, and 50 mL acetone before being dried under vacuum at 60 °C for 2 h. Finally, the black, solid product was obtained after a final annealing step at 400 °C for 2 h.

In order to formulate the screen-printing paste, 1 g of synthesized NiCo_2_O_4_ was dispersed in 40 mL ethanol using an ultrasonic bath for 30 min. Simultaneously, 600 mg ethyl cellulose was dissolved in 10 mL ethanol and the resulting solution was added to the suspension, followed by 7 mL (6538 g) terpineol. The entire mixture was left with agitation overnight using a magnetic stirrer. The following day, the mixture was milled using a ball mill (Across International, PQ-N04) for 4 h at 400 rpm with 30 mL ethanol to facilitate proper blend grinding. Afterward, the mixture was rotary evaporated under vacuum to remove the ethanol and attain the desired rheological properties necessary for successful deposition of homogeneous nanostructured, mesoporous films by screen printing.

### 4.3 C-PSC Device Fabrication

A thin, compact TiO_2_ film was deposited onto precleaned, laser-scribed FTO substrates using spray pyrolysis. A 10% titanium diisopropoxide bis­(acetylacetonate) solution in anhydrous isopropanol was used to spray-deposit onto glass|FTO at 400 °C. After 30 deposition cycles, the substrates were heated to 500 °C for 30 min. Next, *a* mesoporous TiO_2_ layer was deposited by screen printing from GreatCell paste (30 NR-D) diluted with α-terpineol in a 1:1 ratio. The layer was annealed at 530 °C for 30 min. A mesoporous ZrO_2_ layer was also deposited by screen printing from a Solaronix paste (Zr-Nanoxide ZT/SP). Two coats of the paste were deposited to ensure proper thickness, and the layers were annealed at 500 °C for 30 min. For the new architecture, an additional NiCo_2_O_4_ layer was then deposited onto the zirconia layer using our previously prepared paste by screen printing, which was annealed at 500 °C for 30 min. Finally, a carbon layer was deposited from a GEM paste using the same screen printing method and annealed at 400 °C for 30 min. The reference devices were fabricated in the typical triple-stack structure without the NiCo_2_O_4_ layer. After sintering, the substrates were removed at 150 °C and kept in a dry room (RH 30%) up to the infiltration process.

The infiltration process was performed using a drop-cast method. First, the dried and cold substrates were masked with Kapton tape. Next, 23 μL of a freshly prepared 1 M solution of PbI_2_, MAI (97 wt %), and 5-AVAI (3 wt %) in a mixture of γ-valerolactone and methanol (9:1 volume ratio) was dropped onto the mesoporous architecture from the carbon side. The substrates were then left at room temperature for 20 min before being transferred to a hot plate at 45 °C for 1 h, covered with a Petri dish lid. Subsequently, they were uncovered and heated at the same temperature for another 1.5 h to achieve complete conversion. The photovoltaic (PV) devices were stored in the dry room under 30–35% relative humidity.

The performance of C-PSCs has been shown to depend on the time stored under ambient laboratory conditions.^87^ We evaluated the performance of our fabricated devices after 1, 8, 15, and 21 days following infiltration, and found that the best performance was achieved after 15 days for all samples. Hence, this optimal storage time was used for all cells.

### 4.4 Characterization Techniques

A variety of techniques were used to analyze the synthesized NiCo_2_O_4_ powder. XRD analysis was performed using a Bruker D-8 Advance diffractometer with Cu–Kα radiation (40 kV, 30 mA, λ = 1.5418 Å), a step size of 0.02°, and an integration time of 0.5 s. UV–Vis diffuse reflectance spectroscopy (DRS) was conducted using a fiber optics arrangement, with a deuterium-halogen light source (AvaLight DH-S-BAL), an integrating sphere (Labsphere USRS-99-010) interconnected with a spectrometer AvaSpec-2048 from Avantes Co. An Ocean Optics WS-1-SL standard was used as a reference. Vibrational Raman spectroscopy was performed using a Witec spectrometer, α 300RA model with a 488 nm blue laser and a 100x objective lens. Each spectrum was obtained as an average of 32 acquired spectra every 0.5 s. XPS analyses were conducted using a Thermo Scientific equipment, Fischer K-Alpha model, equipped with a monochromatic Al–Kα (1486.7 eV) X-ray source and a spot diameter of 400 μm. To acquire high-resolution spectra, the pass energy employed was 10 eV. The base pressure of the analysis chamber was 10^–9^ Torr for data acquisition. The XPS core-level peaks were deconvoluted into various components using an interactive least-squares program AAnalyzer, 2.04 version.^88^ TGA–DSC analyses were conducted using a TA Instrument apparatus Discovery Series model. The TGA curves were acquired under a 25 mL/min N_2_ flow. SEM images were obtained using a JEOL microscope, JSM-7601F. The images were captured under the LABE (low angle backscattered electrons) scanning mode, where brighter zones indicate atoms with higher atomic numbers and permit clear distinction between the TiO_2_ and ZrO_2_ layers. The specific hexagonal morphology of the larger NiCo_2_O_4_ particles allowed for easy identification of the layer thickness. Prior to solar cell fabrication and characterization, the thickness of the individual layers was optimized and characterized by SEM.

The photovoltaic characteristics of the devices were measured under an ambient atmosphere using an Autolab PGSTAT302 N and a xenon arc lamp, Oriel 66924, equipped with AM 1.5G and water filters. The calibration was done using a Newport-calibrated reference silicon solar cell with an incorporated KG-5 optical filter. The devices were measured from reverse to forward bias (from 1.2 to −0.2 V) and forward to reverse bias (from −0.2 to 1.2 V) at a scan rate of 0.05 V s^–1^. The active area was defined using a shadow mask of 0.16 cm^2^. Before measurement, each device was exposed to 15 min of light soaking. The reverse scan curves were used in the analysis to provide more reliable efficiency values by mitigating transient effects, including those linked to capacitive and ionic phenomena. All *J–V* curves were carefully analyzed to avoid overshoot effects in the calculated photovoltaic parameters, which has been identified as a potential issue in similar systems.^73^ The EIS measurements were carried out using an Autolab/PGSTAT12/30/302N FRA2 system. The applied voltage was equal to the observed *V*_OC_ across a wide range of DC light intensities, using white, blue (480 nm), and red (650 nm) Thorlabs LEDs. A 10 mV perturbation amplitude was applied in the range of 10° to 10^5^ Hz, as no additional signals were detected beyond this range during an initial sweep. The measurements were performed at 25 °C under an inert atmosphere (N_2_) using a 0.25 cm^2^ shadow mask. The data was generated using NOVA 2.1. software, and the Z-view equivalent circuit modeling software (Scribner) was used to fit the spectra.

## Supplementary Material



## Notes

The Supporting Information contains detailed structural and morphological analysis of the NiCo_2_O_4_ material as well as the full devices (TGA; Raman spectroscopy; XRD; UV–vis diffuse reflectance spectroscopy; top view and cross-sectional SEM before and after infiltration). A schematic illustration of the configuration of the C-PSCs fabricated in this work. Three Tables with the photovoltaic parameters of 10 devices each of the reference cells and the cells with 1.1 μm, 2.1 μm NiCo_2_O_4_ interlayers. Forward and reverse current–voltage curves for the champion cells for the three configurations, illustrating the hysteresis. Cole–Cole diagrams from the impedance spectroscopy measurements; the equivalent circuit used to fit the impedance spectroscopy data and a plot of the extracted series resistance as a function of open-circuit voltage; ideality factors determined from the high and low frequency resistance as a function of the LED wavelength (PDF)

## References

[1] Kojima A., Teshima K., Shirai Y., Miyasaka T. (2009). Organometal Halide
Perovskites as Visible-Light Sensitizers for Photovoltaic Cells. J. Am. Chem. Soc..

[2] NREL . Best Research-Cell Efficiency Chart. https://www.nrel.gov/pv/cell-efficiency.html (accessed Jan 23, 2023).

[3] Pourjafari D., Meroni S. M. P., Peralta Domínguez D., Escalante R., Baker J., Saadi Monroy A., Walters A., Watson T., Oskam G. (2022). Strategies towards Cost Reduction in the Manufacture of Printable
Perovskite Solar Modules. Energies.

[4] Jiao B., Ye Y., Tan L., Liu Y., Ren N., Li M., Zhou J., Li H., Chen Y., Li X., Yi C. (2024). Realizing Stable Perovskite
Solar Cells with Efficiency Exceeding
25.6% Through Crystallization Kinetics and Spatial Orientation Regulation. Adv. Mater..

[5] Lan Z., Wang Y., Shao J., Ma D., Liu Z., Li D., Hou Y., Yao J., Zhong Y. (2024). Surface Passivation
with Diaminopropane Dihydroiodide for P-i-n Perovskite Solar Cells
with Over 25% Efficiency. Adv. Funct. Mater..

[6] Rombach F. M., Haque S. A., Macdonald T. J. (2021). Lessons
Learned from Spiro-OMeTAD
and PTAA in Perovskite Solar Cells. Energy Environ.
Sci..

[7] Behrouznejad F., Shahbazi S., Taghavinia N., Wu H.-P., Wei-Guang
Diau E. (2016). A Study on Utilizing Different Metals as the Back Contact of CH_3_NH_3_PbI_3_ Perovskite Solar Cells. J. Mater. Chem. A.

[8] Wagner L., Suo J., Yang B., Bogachuk D., Gervais E., Pietzcker R., Gassmann A., Goldschmidt J. C. (2024). The Resource Demands of Multi-Terawatt-Scale
Perovskite Tandem Photovoltaics. Joule.

[9] Jena A. K., Numata Y., Ikegami M., Miyasaka T. (2018). Role of Spiro-OMeTAD
in Performance Deterioration of Perovskite Solar Cells at High Temperature
and Reuse of the Perovskite Films to Avoid Pb-Waste. J. Mater. Chem. A.

[10] Liang L., Cai Y., Li X., Nazeeruddin M. K., Gao P. (2018). All That Glitters Is
Not Gold: Recent Progress of Alternative Counter Electrodes for Perovskite
Solar Cells. Nano Energy.

[11] Thiesbrummel, J. ; Shah, S. ; Gutierrez-Partida, E. ; Zu, F. ; Peña-Camargo, F. ; Zeiske, S. ; Diekmann, J. ; Ye, F. ; Peters, K. P. ; Brinkmann, K. O. ; Caprioglio, P. ; Dasgupta, A. ; Seo, S. ; Adeleye, F. A. ; Warby, J. ; Jeangros, Q. ; Lang, F. ; Zhang, S. ; Albrecht, S. ; Riedl, T. ; Armin, A. ; Neher, D. ; Koch, N. ; Wu, Y. ; Le Corre, V. M. ; Snaith, H. ; Stolterfoht, M. Ion-Induced Field Screening as a Dominant Factor in Perovskite Solar Cell Operational Stability. Nat. Energy 2024. 9 664 10.1038/s41560-024-01487-w.

[12] Islam M. B., Yanagida M., Shirai Y., Nabetani Y., Miyano K. (2019). Highly Stable
Semi-Transparent MAPbI_3_ Perovskite Solar Cells with Operational
Output for 4000 h. Sol. Energy Mater.
Sol. Cells..

[13] Le T. S., Saranin D., Gostishchev P., Ermanova I., Komaricheva T., Luchnikov L., Muratov D., Uvarov A., Vyacheslavova E., Mukhin I., Didenko S., Kuznetsov D., Di Carlo A. (2022). All-Slot-Die-Coated Inverted Perovskite Solar Cells
in Ambient Conditions with Chlorine Additives. Solar RRL.

[14] Tan W., Xie C., Liu Y., Zhao Y., Li L., Liu X., Yuan Y., Li Y., Gao Y. (2018). Initial Photochemical
Stability in Perovskite Solar Cells Based on the Cu Electrode and
the Appropriate Charge Transport Layers. Synth.
Met..

[15] Chen R., Wang J., Liu Z., Ren F., Liu S., Zhou J., Wang H., Meng X., Zhang Z., Guan X., Liang W., Troshin P. A., Qi Y., Han L., Chen W. (2023). Reduction of Bulk and Surface Defects in Inverted Methylammonium-
and Bromide-Free Formamidinium Perovskite Solar Cells. Nat. Energy.

[16] Kang X., Wang D., Sun K., Dong X., Hui W., Wang B., Gu L., Li M., Bao Y., Zhang J., Guo R., Li Z., Jiang X., Müller-Buschbaum P., Song L. (2023). Unraveling the Modification
Effect at NiO_
*x*
_ /Perovskite Interfaces
for Efficient and Stable Inverted Perovskite Solar Cells. J. Mater. Chem. A.

[17] Liu Y., Duan J., Zhang J., Huang S., Ou-Yang W., Bao Q., Sun Z., Chen X. (2020). High Efficiency and Stability of
Inverted Perovskite Solar Cells Using Phenethyl Ammonium Iodide-Modified
Interface of NiO_x_ and Perovskite Layers. ACS Appl. Mater. Interfaces.

[18] Mohanraj J., Samanta B., Almora O., Escalante R., Marsal L. F., Jenatsch S., Gadola A., Ruhstaller B., Anta J. A., Caspary Toroker M., Olthof S. (2024). NiO_X_ Passivation
in Perovskite Solar Cells: From Surface Reactivity to Device Performance. ACS Appl. Mater. Interfaces.

[19] Chen P., Xiao Y., Li S., Jia X., Luo D., Zhang W., Snaith H. J., Gong Q., Zhu R. (2024). The Promise
and Challenges of Inverted Perovskite Solar Cells. Chem. Rev..

[20] Ku Z., Rong Y., Xu M., Liu T., Han H. (2013). Full Printable
Processed Mesoscopic CH_3_NH_3_PbI_3_/TiO_2_ Heterojunction Solar Cells with Carbon Counter Electrode. Sci. Rep..

[21] Mei A., Li X., Liu L., Ku Z., Liu T., Rong Y., Xu M., Hu M., Chen J., Yang Y., Grätzel M., Han H. (2014). A Hole-Conductor–Free, Fully Printable Mesoscopic Perovskite
Solar Cell with High Stability. Science.

[22] Baker J., Hooper K., Meroni S., Pockett A., McGettrick J., Wei Z., Escalante R., Oskam G., Carnie M., Watson T. (2017). High Throughput
Fabrication of Mesoporous Carbon Perovskite Solar Cells. J. Mater. Chem. A.

[23] Pourjafari D., García-Peña N. G., Padrón-Hernández W. Y., Peralta-Domínguez D., Castro-Chong A. M., Nabil M., Avilés-Betanzos R. C., Oskam G. (2023). Functional
Materials for Fabrication of Carbon-Based Perovskite Solar Cells:
Ink Formulation and Its Effect on Solar Cell Performance. Materials.

[24] Bogachuk D., Saddedine K., Martineau D., Narbey S., Verma A., Gebhardt P., Herterich J. P., Glissmann N., Zouhair S., Markert J., Gould I. E., McGehee M. D., Würfel U., Hinsch A., Wagner L. (2022). Perovskite Photovoltaic
Devices with Carbon-Based Electrodes Withstanding Reverse-Bias Voltages
up to – 9 V and Surpassing IEC 61215:2016 International Standard. Solar RRL.

[25] Hu Y., Si S., Mei A., Rong Y., Liu H., Li X., Han H. (2017). Stable Large-Area
(10 × 10 cm^2^) Printable Mesoscopic
Perovskite Module Exceeding 10% Efficiency. Solar RRL.

[26] Grancini G., Roldán-Carmona C., Zimmermann I., Mosconi E., Lee X., Martineau D., Narbey S., Oswald F., De Angelis F., Graetzel M., Nazeeruddin M. K. (2017). One-Year Stable Perovskite Solar
Cells by 2D/3D Interface Engineering. Nat. Commun..

[27] Caprioglio P., Stolterfoht M., Wolff C. M., Unold T., Rech B., Albrecht S., Neher D. (2019). On the Relation between the Open-Circuit
Voltage and Quasi-Fermi Level Splitting in Efficient Perovskite Solar
Cells. Adv. Energy Mater..

[28] Chen H., Yang S. (2019). Methods and Strategies for Achieving High-Performance Carbon-Based
Perovskite Solar Cells without Hole Transport Materials. J. Mater. Chem. A.

[29] Liu S., Huang W., Liao P., Pootrakulchote N., Li H., Lu J., Li J., Huang F., Shai X., Zhao X., Shen Y., Cheng Y.-B., Wang M. (2017). 17% Efficient
Printable Mesoscopic PIN Metal Oxide Framework Perovskite Solar Cells
Using Cesium-Containing Triple Cation Perovskite. J. Mater. Chem. A.

[30] Peiris T. A. N., Baranwal A. K., Kanda H., Fukumoto S., Kanaya S., Cojocaru L., Bessho T., Miyasaka T., Segawa H., Ito S. (2017). Enhancement of the
Hole Conducting Effect of NiO by a N_2_ Blow Drying Method
in Printable Perovskite Solar Cells with Low-Temperature
Carbon as the Counter Electrode. Nanoscale.

[31] Bashir A., Shukla S., Lew J. H., Shukla S., Bruno A., Gupta D., Baikie T., Patidar R., Akhter Z., Priyadarshi A., Mathews N., Mhaisalkar S. G. (2018). Spinel
Co_3_O_4_ Nanomaterials for Efficient and Stable
Large Area Carbon-Based Printed Perovskite Solar Cells. Nanoscale.

[32] Chen K., Xiao X., Liu J., Qi J., Gao Q., Ma Y., Cheng Y., Mei A., Han H. (2024). Record-Efficiency Printable
Hole-Conductor-Free Mesoscopic Perovskite Solar Cells Enabled by the
Multifunctional Schiff Base Derivative. Adv.
Mater..

[33] Liu J., Chen X., Chen K., Tian W., Sheng Y., She B., Jiang Y., Zhang D., Liu Y., Qi J., Chen K., Ma Y., Qiu Z., Wang C., Yin Y., Zhao S., Leng J., Jin S., Zhao W., Qin Y., Su Y., Li X., Li X., Zhou Y., Zhou Y., Ling F., Mei A., Han H. (2024). Electron Injection
and Defect Passivation for High-Efficiency Mesoporous Perovskite Solar
Cells. Science.

[34] Wu C., liu L., Xu R., Zhang K., Zhen C., Ma L., Hou D. (2021). Theoretical
Analysis on O Vacancy and Cation Inversion in NiCo_2_O_4_. Mater. Sci. Eng., B.

[35] Wu Z., Zhu Y., Ji X. (2014). NiCo_2_O_4_-Based Materials for Electrochemical
Supercapacitors. J. Mater. Chem. A.

[36] Sun D. S., Li Y. H., Wang Z. Y., Cheng X. P., Jaffer S., Zhang Y. F. (2016). Understanding the
Mechanism of Hydrogenated NiCo_2_O_4_ Nanograss
Supported on Ni Foam for Enhanced-Performance
Supercapacitors. J. Mater. Chem. A.

[37] Bacelis-Martínez R. D., Oskam G., Rodriguez Gattorno G., Ruiz-Gómez M. A. (2017). Inkjet
Printing as High-Throughput Technique for the Fabrication of NiCo_2_O_4_ Films. Adv. Mater. Sci.
Eng..

[38] McCloy J. S., Jiang W., Bennett W., Engelhard M., Lindemuth J., Parmar N., Exarhos G. J. (2015). Electrical and Magnetic
Properties Modification in Heavy Ion Irradiated Nanograin Ni_
*x*
_Co_(3–*x*)_O_4_ Films. J. Phys. Chem. C.

[39] Kan D., Suzuki I., Shimakawa Y. (2020). Influence
of Deposition Rate on Magnetic
Properties of Inverse-Spinel NiCo_2_O_4_ Epitaxial
Thin Films Grown by Pulsed Laser Deposition. Jpn. J. Appl. Phys..

[40] Pore O. C., Fulari A. V., Chavare C. D., Sawant D. S., Patil S. S., Shejwal R. V., Fulari V. J., Lohar G. M. (2023). Synthesis
of NiCo_2_O_4_ Microflowers by Facile Hydrothermal
Method:
Effect of Precursor Concentration. Chem. Phys.
Lett..

[41] Guan X.-H., Li M., Zhang H.-Z., Yang L., Wang G.-S. (2018). Template-Assisted
Synthesis of NiCoO_2_ Nanocages/Reduced Graphene Oxide Composites
as High-Performance Electrodes for Supercapacitors. RSC Adv..

[42] Kaur M., Chand P., Anand H. (2022). Facile Synthesis
of NiCo_2_O_4_ Nanostructure with Enhanced Electrochemical
Performance
for Supercapacitor Application. Chem. Phys.
Lett..

[43] Liu Y., Wang N., Yang C., Hu W. (2016). Sol–Gel Synthesis
of Nanoporous NiCo_2_O_4_ Thin Films on ITO Glass
as High-Performance Supercapacitor Electrodes. Ceram. Int..

[44] Hagen D. J., Tripathi T. S., Karppinen M. (2017). Atomic Layer
Deposition of Nickel–Cobalt
Spinel Thin Films. Dalton Trans..

[45] Kaur M., Chand P., Anand H. (2022). Binder Free
Electrodeposition Fabrication
of NiCo_2_O_4_ Electrode with Improved Electrochemical
Behavior for Supercapacitor Application. J.
Energy Storage.

[46] Deokate R. J., Kalubarme R. S., Park C.-J., Lokhande C. D. (2017). Simple
Synthesis
of NiCo_2_O_4_ Thin Films Using Spray Pyrolysis
for Electrochemical Supercapacitor Application: A Novel Approach. Electrochim. Acta.

[47] Waghmode R. B., Maile N. C., Lee D. S., Torane A. P. (2020). Chemical Bath Synthesis
of NiCo_2_O_4_ Nanoflowers with Nanorods like Thin
Film for Flexible Supercapacitor Application-Effect of Urea Concentration
on Structural Conversion. Electrochim. Acta.

[48] Bashir A., Shukla S., Bashir R., Patidar R., Bruno A., Gupta D., Satti M. S., Akhter Z. (2020). Low Temperature, Solution
Processed Spinel NiCo_2_O_4_ Nanoparticles as Efficient
Hole Transporting Material for Mesoscopic n-i-p Perovskite Solar Cells. Sol. Energy.

[49] Jeong J.-Y., Park Y. S., Jeong J., Lee K.-B., Kim D., Yoon K.-Y., Park H.-S., Yang J. A. (2022). NiCo_2_O_4_ Electrocatalyst with a Thin Graphitic Coating for the
Anion Exchange Membrane Water Electrolysis of Wastewater. J. Mater. Chem. A.

[50] Zhang L., Li Y., Peng J., Peng K. (2019). Bifunctional NiCo_2_O_4_ Porous Nanotubes Electrocatalyst
for Overall Water-Splitting. Electrochim. Acta.

[51] Wang C., Zhou E., He W., Deng X., Huang J., Ding M., Wei X., Liu X., Xu X. (2017). NiCo_2_O_4_-Based Supercapacitor
Nanomaterials. Nanomaterials.

[52] Kumar L., Chauhan H., Yadav N., Yadav N., Hashmi S. A., Deka S. (2018). Faster Ion Switching
NiCo_2_O_4_ Nanoparticle Electrode-Based
Supercapacitor Device with High Performances and Long Cycling Stability. ACS Appl. Energy Mater..

[53] Li D., Gong Y., Zhang Y., Luo C., Li W., Fu Q., Pan C. (2015). Facile Synthesis of
Carbon Nanosphere/NiCo_2_O_4_ Core-Shell Sub-Microspheres
for High Performance Supercapacitor. Sci. Rep..

[54] Du J., Zhou G., Zhang H., Cheng C., Ma J., Wei W., Chen L., Wang T. (2013). Ultrathin Porous NiCo_2_O_4_ Nanosheet Arrays on
Flexible Carbon Fabric for High-Performance
Supercapacitors. ACS Appl. Mater. Interfaces.

[55] Marimuthu G., Palanisamy G., Pazhanivel T., Bharathi G., Cristopher M. M., Jeyadheepan K. (2020). Nanorod like NiCo_2_O_4_ Nanostructure
for High Sensitive and Selective Ammonia Gas Sensor. J. Mater. Sci.: Mater. Electron..

[56] Mahboubi H., Masoudpanah S. M., Alamolhoda S., Hasheminiasari M. (2023). Facile Synthesis
of Spongy NiCo_2_O_4_ Powders for Lithium-Ion Storage. Sci. Rep..

[57] Zhang C., Deng L., Zhang P., Ren X., Li Y., He T. (2017). Mesoporous NiCo_2_O_4_ Networks with
Enhanced Performance
as Counter Electrodes for Dye-Sensitized Solar Cells. Dalton Trans..

[58] Li L., Ma Z., Liu L., Wang X., Wang J., Cao L., Liu S., Zhang W. (2022). NiCo_2_O_4_/Carbon Nanofibers Composite
as an Efficient Counter Electrode for Dye-Sensitized Solar Cells. Mater. Res. Bull..

[59] Shi Z., Lu H., Liu Q., Deng K., Xu L., Zou R., Hu J., Bando Y., Golberg D., Li L. (2014). NiCo_2_O_4_ Nanostructures as a Promising Alternative for NiO Photocathodes
in P-Type Dye-Sensitized Solar Cells with High Efficiency. Energy Technol..

[60] Mottakin M., Sarkar D. K., Selvanathan V., Rashid M. J., Sobayel K., Hasan A. K. M., Md A. I., Muhammad G., Md S., Md A. (2023). Photoelectric Performance
of Environmentally Benign Cs_2_TiBr_6_-Based Perovskite
Solar Cell Using Spinel NiCo_2_O_4_ as HTL. Optik.

[61] Wang S., Wang L., Liu C., Shan Y., Li F., Sun L. (2022). NiCo_2_O_4_ Thin Film Prepared by Electrochemical
Deposition as a Hole-Transport Layer for Efficient Inverted Perovskite
Solar Cells. RSC Adv..

[62] Venkatachalam V., Alsalme A., Alghamdi A., Jayavel R. (2015). High Performance Electrochemical
Capacitor Based on MnCo_2_O_4_ Nanostructured Electrode. J. Electroanal. Chem..

[63] Venkatachalam V., Alsalme A., Alghamdi A., Jayavel R. (2017). Hexagonal-like NiCo_2_O_4_ Nanostructure
Based High-Performance Supercapacitor
Electrodes. Ionics.

[64] Mcintyre N. S., Cook M. G. (1975). X-Ray Photoelectron
Studies on Some Oxides and Hydroxides
of Cobalt, Nickel, and Copper. Anal. Chem..

[65] Hagelin-Weaver H. A.
E., Hoflund G. B., Minahan D. M., Salaita G. N. (2004). Electron Energy
Loss Spectroscopic Investigation of Co Metal, CoO, and Co_3_O_4_ before and after Ar^+^ Bombardment. Appl. Surf. Sci..

[66] Manalu A., Tarigan K., Humaidi S., Ginting M., Sebayang K., Rianna M., Hamid M., Subhan A., Sebayang P., Manalu I. P. (2022). Synthesis, Microstructure
and Electrical Properties
of NiCo_2_O_4_/RGO Composites as Pseudocapacitive
Electrode for Supercapacitors. Int. J. Electrochem.
Sci..

[67] Cui B., Lin H., Liu Y. Z., Li J. B., Sun P., Zhao X. C., Liu C. J. (2009). Photophysical and Photocatalytic Properties of Core-Ring
Structured NiCo_2_O_4_ Nanoplatelets. J. Phys. Chem. C.

[68] Jia C., Yang F., Zhao L., Cheng G., Yang G. (2019). Temperature-Dependent
Electrical Transport Properties of Individual NiCo_2_O_4_ Nanowire. Nanoscale Res. Lett..

[69] Correa-Baena J., Anaya M., Lozano G., Tress W., Domanski K., Saliba M., Matsui T., Jacobsson T. J., Calvo M. E., Abate A., Grätzel M., Míguez H., Hagfeldt A. (2016). Unbroken Perovskite: Interplay of
Morphology, Electro-optical Properties, and Ionic Movement. Adv. Mater..

[70] Almora O., Lopez-Varo P., Cho K. T., Aghazada S., Meng W., Hou Y., Echeverría-Arrondo C., Zimmermann I., Matt G. J., Jiménez-Tejada J. A., Brabec C. J., Nazeeruddin M. K., Garcia-Belmonte G. (2019). Ionic Dipolar Switching Hinders Charge
Collection in Perovskite Solar Cells with Normal and Inverted Hysteresis. Sol. Energy Mater. Sol. Cells.

[71] Phung N., Al-Ashouri A., Meloni S., Mattoni A., Albrecht S., Unger E. L., Merdasa A., Abate A. (2020). The Role of Grain Boundaries
on Ionic Defect Migration in Metal Halide Perovskites. Adv. Energy Mater..

[72] García-Rodríguez R., Riquelme A. J., Cowley M., Valadez-Villalobos K., Oskam G., Bennett L. J., Wolf M. J., Contreras-Bernal L., Cameron P. J., Walker A. B., Anta J. A. (2022). Inverted Hysteresis
in n–i–p and p–i–n Perovskite Solar Cells. Energy Technol..

[73] De
Moor G., Charvin N., Farha C., Meyer T., Perrin L., Planes E., Flandin L. (2024). Understanding the Anomalous *J–V* Curves in Carbon-Based Perovskite Solar Cells
as a Structural Transition Induced by Ion Diffusion. Solar RRL.

[74] Riquelme A. J., Valadez-Villalobos K., Boix P. P., Oskam G., Mora-Seró I., Anta J. A. (2022). Understanding Equivalent Circuits in Perovskite Solar
Cells. Insights from Drift-Diffusion Simulation. Phys. Chem. Chem. Phys..

[75] Pockett A., Eperon G. E., Peltola T., Snaith H. J., Walker A., Peter L. M., Cameron P. J. (2015). Characterization
of Planar Lead Halide
Perovskite Solar Cells by Impedance Spectroscopy, Open-Circuit Photovoltage
Decay, and Intensity-Modulated Photovoltage/Photocurrent Spectroscopy. J. Phys. Chem. C.

[76] Zarazúa I., Sidhik S., Lopéz-Luke T., Esparza D., De La Rosa E., Reyes-Gomez J., Mora-Seró I., Garcia-Belmonte G. (2017). Operating
Mechanisms of Mesoscopic Perovskite Solar Cells through Impedance
Spectroscopy and J-V Modeling. J. Phys. Chem.
Lett..

[77] Bou A., Pockett A., Raptis D., Watson T., Carnie M. J., Bisquert J. (2020). Beyond Impedance Spectroscopy of Perovskite Solar Cells:
Insights from the Spectral Correlation of the Electrooptical Frequency
Techniques. J. Phys. Chem. Lett..

[78] Contreras-Bernal L., Ramos-Terrón S., Riquelme A., Boix P. P., Idígoras J., Mora-Seró I., Anta J. A. (2019). Impedance Analysis of Perovskite
Solar Cells: A Case Study. J. Mater. Chem. A.

[79] Riquelme A., Bennett L. J., Courtier N. E., Wolf M. J., Contreras-Bernal L., Walker A. B., Richardson G., Anta J. A. (2020). Identification of
Recombination Losses and Charge Collection Efficiency in a Perovskite
Solar Cell by Comparing Impedance Response to a Drift-Diffusion Model. Nanoscale.

[80] Contreras-Bernal L., Salado M., Todinova A., Calio L., Ahmad S., Idígoras J., Anta J. A. (2017). Origin and Whereabouts of Recombination
in Perovskite Solar Cells. J. Phys. Chem. C.

[81] Gagliardi A., Abate A. (2018). Mesoporous Electron-Selective Contacts Enhance the Tolerance to Interfacial
Ion Accumulation in Perovskite Solar Cells. ACS Energy Lett..

[82] Zarazua I., Han G., Boix P. P., Mhaisalkar S., Fabregat-Santiago F., Mora-Seró I., Bisquert J., Garcia-Belmonte G. (2016). Surface Recombination
and Collection Efficiency in Perovskite Solar Cells from Impedance
Analysis. J. Phys. Chem. Lett..

[83] Kerremans R., Sandberg O. J., Meroni S., Watson T., Armin A., Meredith P. (2020). On the Electro-Optics of Carbon Stack Perovskite Solar
Cells. Solar RRL.

[84] Zhou L., Zuo Y., Mallick T. K., Sundaram S. (2019). Enhanced Efficiency of Carbon-Based
Mesoscopic Perovskite Solar Cells through a Tungsten Oxide Nanoparticle
Additive in the Carbon Electrode. Sci. Rep..

[85] Clarke W., Cameron P., Richardson G. (2024). Predicting
Long-Term Stability from
Short-Term Measurement: Insights from Modeling Degradation in Perovskite
Solar Cells during Voltage Scans and Impedance Spectroscopy. J. Phys. Chem. Lett..

[86] Madito M. J. (2021). Correlation
of the Graphene Fermi-Level Shift and the Enhanced Electrochemical
Performance of Graphene-Manganese Phosphate for Hybrid Supercapacitors:
Raman Spectroscopy Analysis. ACS Appl. Mater.
Interfaces.

[87] Worsley C., Raptis D., Meroni S., Doolin A., Garcia-Rodriguez R., Davies M., Watson T. (2021). γ-Valerolactone: A Nontoxic
Green Solvent for Highly Stable Printed Mesoporous Perovskite Solar
Cells. Energy Technol..

[88] Herrera-Gomez A. (2020). Uncertainties
in Photoemission Peak Fitting Accounting for the Covariance with Background
Parameters. J. Vac. Sci. Technol. A.

