# Supplementary material for: Improving the Performance of Carbon-Based Perovskite Solar Cells by the Incorporation of a Screen-Printed NiCo2O4 Interlayer
Source: ACS Appl Energy Mater. 2025 Jan 16;8(3):1446–57. doi: 10.1021/acsaem.4c01720 (PMC12691577; doi:10.1021/acsaem.4c01720)
Supplement: Supplementary file 1 [file ae4c01720_si_001.pdf]

Improving the Performance of Carbon-Based Perovskite Solar Cells by Incorporation of a Screen-Printed $NiCo_2O_4$ Interlayer

Nidia G. García-Peña[1,†], Mahmoud Nabil[2,†,], Dena Pourjafari[1], Diecenia Peralta-Domínguez[1], Wendy Yaznay Padrón-Hernández[3], Adriana P. Franco-Bacca[1], Araceli Ríos-Flores[1], Beatriz Eugenia Heredia-Cervera[1,2], Renan Escalante[2], Geonel Rodríguez Gattorno[1], Milenis Acosta[4], Paul Pistor[2], Juan Antonio Anta[2], and Gerko Oskam[1,2,*]*

[1] Department of Applied Physics, CINVESTAV-IPN, Antigua Carretera a Progreso Km 6, Merida 97310, Yucatan, Mexico.
[2] Center for Nanoscience and Sustainable Technologies (CNATS), Department of Physical, Chemical and Natural Systems, Universidad Pablo de Olavide, Carretera de Utrera Km 1, 41013 Seville, Spain.
[3] Facultad de Ingeniería Química, Universidad Autónoma de Yucatán, Periférico Norte, Kilómetro 33.5, Tablaje Catastral 13615, Chuburná de Hidalgo Inn. C.P. 97203. Merida Yucatan, Mexico.
[4] Facultad de Ingeniería, Universidad Autónoma de Yucatán, Avenida Industrias No Contaminantes por Anillo Periférico Norte, Merida 97203, Yucatan, Mexico.

† Both authors contributed equally to this work.
* Corresponding Authors: *mnmah@upo.es* (M. Nabil)
gosk@upo.es (G. Oskam)

SUPPORTING INFORMATION

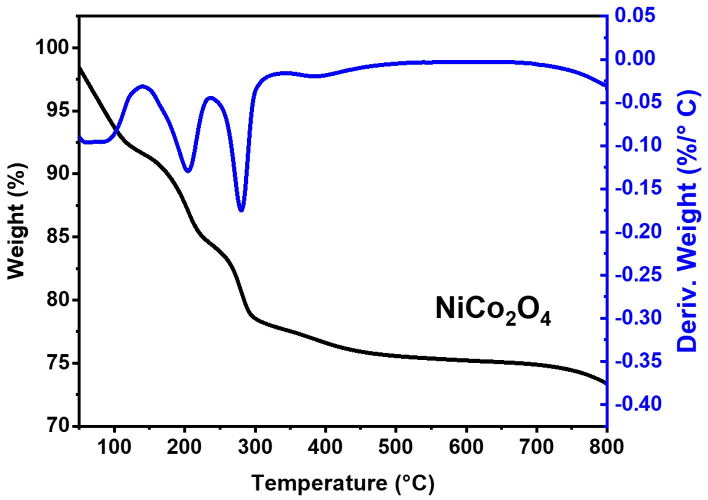

Figure S1. Thermogravimetry analysis of $NiCo_2O_4$.

TGA-DSC analysis was performed on the dried brown co-precipitated solid powder to determine the temperature for complete dehydration and obtain the desired product. The powder was heated from room temperature to 800 °C at a speed of 10 °C/min in air. The resulting thermogram (Figure

S1) showed continued weight losses below 400 °C, indicating water losses during the $NiCo_2O_4$ formation. At 400°C, the powder´s color changed to black, and no significant weight loss was observed from the thermogram above this temperature. At 750 °C, an additional weight loss was observed, possibly indicating oxygen loss and O vacancy formation.[1] Based on these results, the annealing step was carried out at 400 °C for 2 hours to obtain the desired ternary oxide product.

XRD analysis was performed on the final product. The resulting crystallographic pattern from the XRD analysis matched with JCPDS card number 01-073-1702 as shown in Figure S2. The crystal structure is a spinel and corresponds to a triclinic space group (space group P1) with the following lattice parameters: a = 5.815 A, b = 5.823 A, c = 8.153, $\alpha = \beta = \gamma = 90°$. The pattern shows the characteristic peaks at 18.72°, 30.72°, 36.46°, 37.94°, 43.97°, 54.71°, 58.75°, and 63.90° 2-theta degrees, which correspond to (011), (002), (200), (103), (022), (220), (312), (015), and (040) crystal planes, respectively. No other characteristic peak was observed, indicating that high-purity $NiCo_2O_4$ was formed without any impurities or incomplete Ni^{2+} incorporation into the spinel phase.

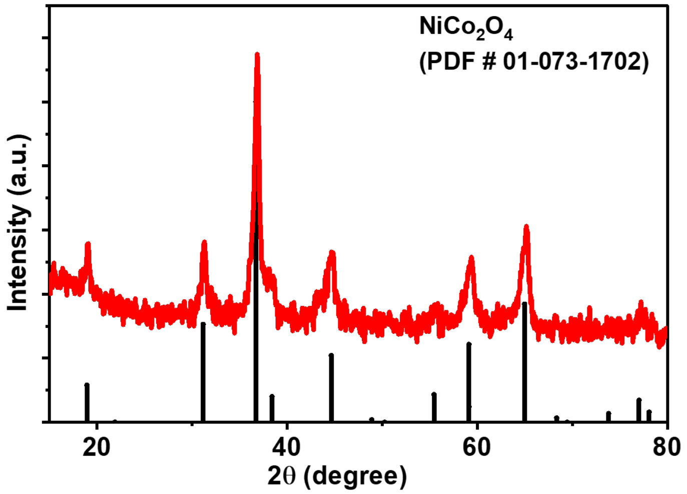

Figure S2. Experimental X-ray diffraction pattern (in red) of the dark product obtained by co-precipitation in comparison with PDF # 01-073-1702 card (lines in black).

Figure S3 shows the Raman spectrum of the product: five signals at 203, 486, 532, 631, and 691 cm^{-1} were observed in the 100 cm^{-1} to 1000 cm^{-1} range. According to the literature, these signals correspond to F_{2g}, E_g, F_{2g}, F_{2g}, and A_{1g} Raman-active modes from MCo_2O_4 spinel structures.[2,3] No other signals were detected when the laser power was below 10 mW. However, higher power

incidence revealed signals beyond 1000 cm^{-1}, which might come from the decomposition of ternary oxide.

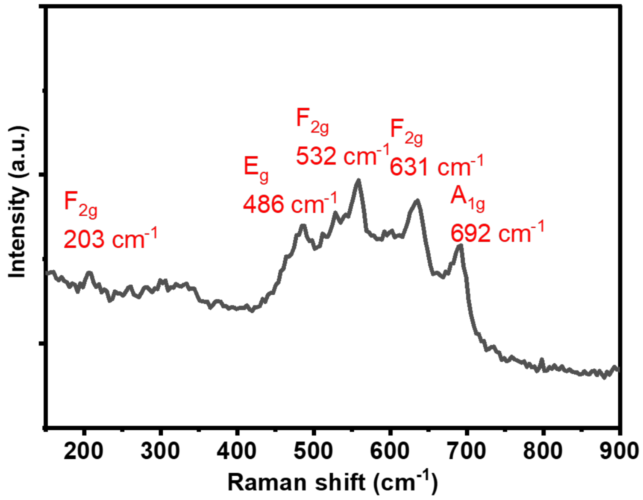

Figure S3. Raman spectroscopy on the $NiCo_2O_4$ powder obtained from the co-precipitation method. The spectrum exhibits the typical five Raman-active modes A_{1g}, E_g, and $3F_{2g}$.

The $NiCo_2O_4$ powder was analysed using XPS, and the results are presented in Figure S4. The survey analysis (Figure S4a) shows signals from C, O, Co, and Ni, along with small quantities of Sn from the sample mount. This result confirms that a pure compound phase, suitable for solar cell applications, was obtained from the synthesis. High-resolution analyses were then conducted. Figure S4b displays the spectrum from the C 1s zone, where the fitted spectrum (red line) was obtained from the experimental result (black dotted line). The spectrum consists of three peaks at 284.8, 285.87, and 288.39 eV (labelled in blue, green, and orange lines in Figure S4b, respectively). The first peak, which comprises most of the spectrum, can be assigned to typical C sp3. This peak is always present in XPS samples that are not eroded[4] and was used to calibrate the high-resolution signals to compensate for the charging effect. The second peak was attributed to acetone traces still present despite the heating treatment, which resulted in C sp3 next to a carbonyl functional group.[5] Finally, the last peak was ascribed to C=O coming from acetone traces.[6]

The high-resolution spectrum in the O 1s zone is shown in Figure S4c. The black dotted line represents the experimental result, and the red line is the fitted spectrum. Four signals could be observed in the spectrum. The first peak, represented by the blue line, is at 529.43 eV. This peak may have originated from oxygen coordinated to nickel in a spinel-like structure.[7] The second

peak, represented by the green line, is at 529.71 eV. This peak can be attributed to a component of the Co^{2+}-O bond in the tetrahedral coordination site in the spinel structure.[8] It is worth noting that Co^{2+} may persist despite H_2O_2 oxidation. Another possibility is the presence of Co^{3+} in a tetrahedral site,[8] which exists in the $NiCo_2O_4$ oxide. The third peak, represented by the orange line, is at 531.03 eV. This peak arises from oxygen attached to Co^{3+} in an octahedral geometry.[9] The fourth signal at 532.72 eV, represented by the purple line, could be assigned to residual water in the sample.[10]

In the Co 2p zone (Figure S4d), the fitted spectrum (in red) was analysed and identified as three doublet signals. The first signal (blue line) at 794.36 and 779.31 eV is most likely Co^{n+}-O in a tetrahedral geometry, which has been previously detected in the O 1s zone.[11] The second doublet (green line) at 780.50 and 795.86 eV originates from Co^{3+}-O in an octahedral site.[11] Finally, the broad signal (orange line) at 789.71 and 803.58 eV is a satellite signal from high spin states.[11]

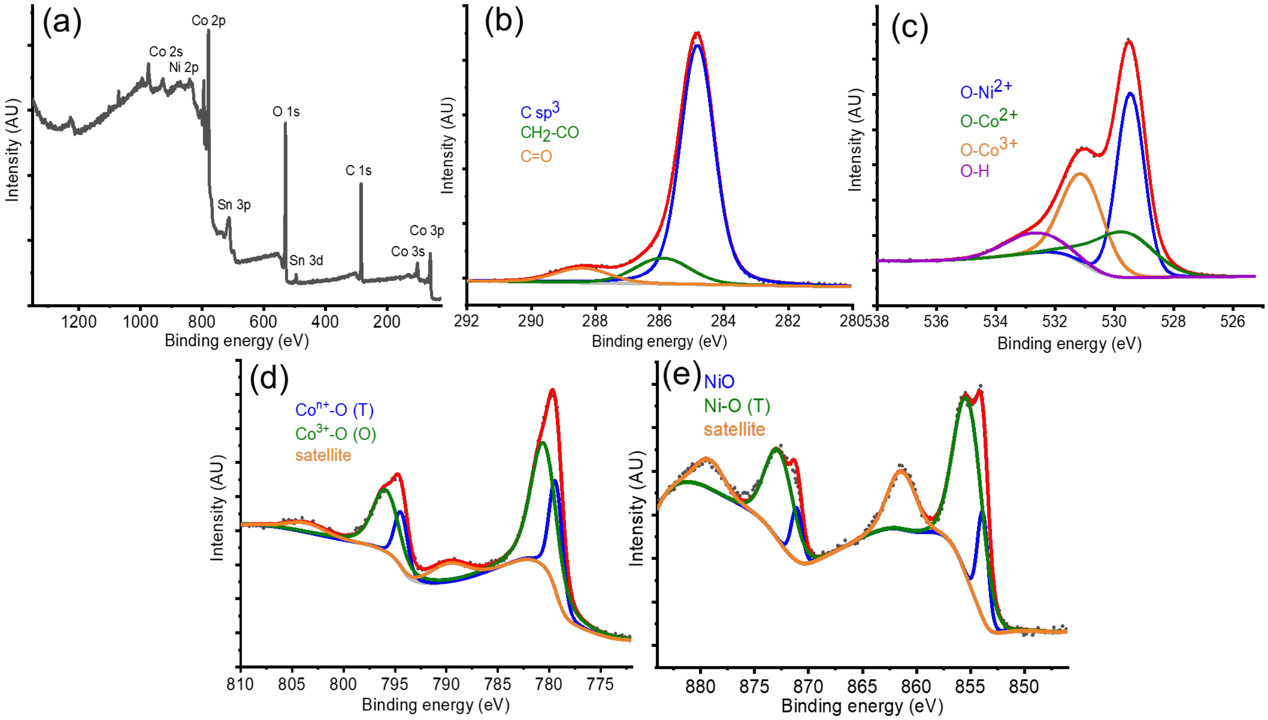

Figure S4. X-ray photoelectron spectroscopy analysis of $NiCo_2O_4$.

Likewise, three signals were observed in the Ni 2p zone (Figure S4e). The first signal is a doublet at 853.81 and 871.06 eV (blue line), which may indicate the presence of a small concentration of

NiO on the surface of the crystallites.[12,13] This signal corresponds to approximately 10% of the spectrum. The second signal is a doublet at 855.16 and 872.67 eV (green line), which agrees with Ni-O in a tetrahedral coordination site in a spinel MN_2O_4 structure.[8] The final one is a broad twin signal at 861.33 and 879.12 eV (orange line), arising from satellite peaks from paramagnetic Ni^{2+} [7].

UV-Vis diffuse reflectance spectroscopy was performed to analyse the powder sample. Figure S5a illustrates the obtained spectrum. The Kubelka-Munk function was calculated from this spectrum and plotted against photon energy, as shown in Figure S5b. Two direct band gaps were identified at 1.98 eV and 2.53 eV. The former corresponds to the usual reported value for this compound[14] while the latter was unexpected but consistent with previous studies of $NiCo_2O_4$ hexagonal nanoplatelets.[15] The co-existence of Co^{2+} and Co^{3+} in high-spin and low-spin states in these structures was suggested as the reason for the two band gaps.[16] This result agrees with the spinel structure of $NiCo_2O_4$, where Co^{3+} can be found in both tetrahedral (high-spin) and octahedral (low-spin) coordination sites. The presence of small amounts of Co^{2+} incorporated into tetrahedral sites was also suggested as a possible explanation for the observed results since the oxide was synthesized from a Co^{2+} salt oxidized with H_2O_2.

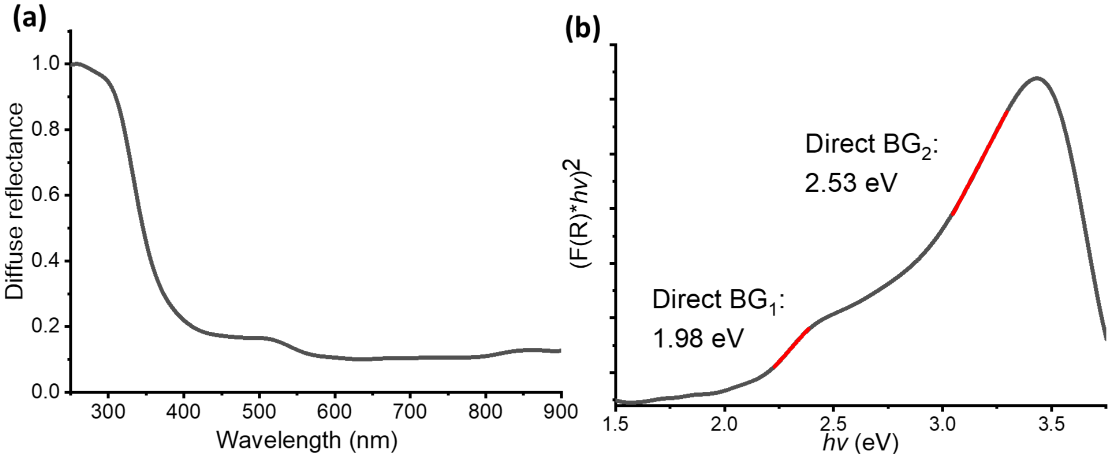

Figure S5. (a) UV-vis electronic diffuse reflectance spectroscopy of $NiCo_2O_4$ product synthesized by co-precipitation method. (b) And Kubelka-Munk function obtained from diffuse reflectance spectroscopy, where two direct band gaps at 1.98 eV and 2.53 eV were calculated.

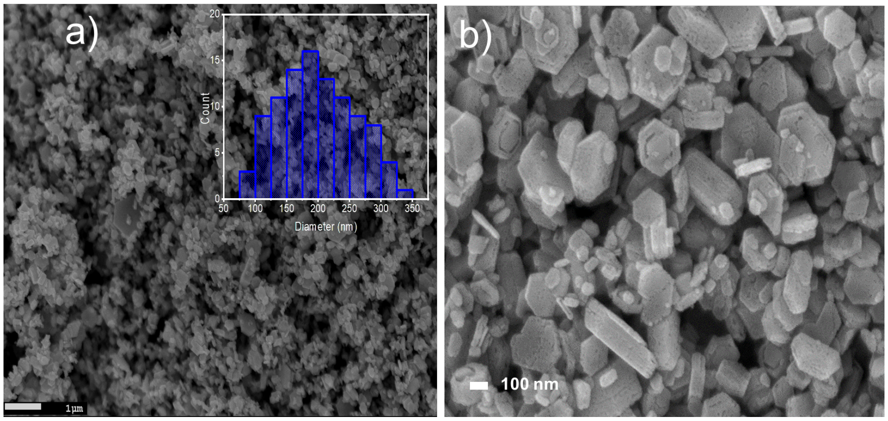

Figure S6. Scanning electron micrographs of $NiCo_2O_4$ oxide.

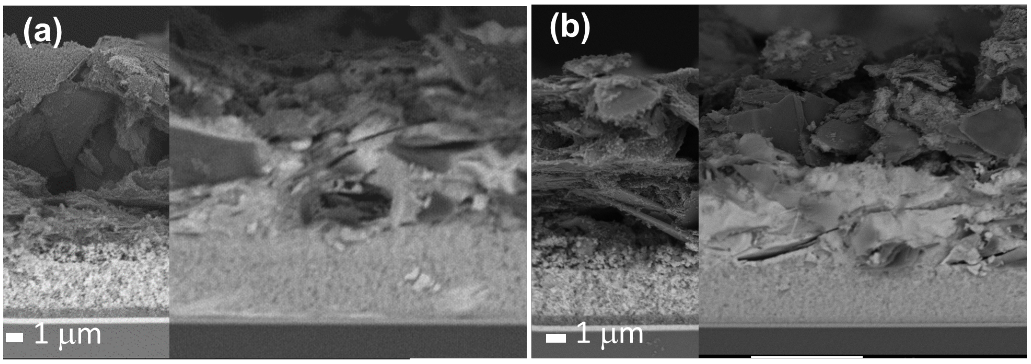

Figure S7. Cross-sectional SEM micrographs of the screen-printed mesoporous stacks with the $NiCo_2O_4$ interlayer: (a) 2.1 µm; from the original paste; and (b) 1.1 µm: from a diluted paste. Images are shown for both before (left side) and after (right side) perovskite infiltration.

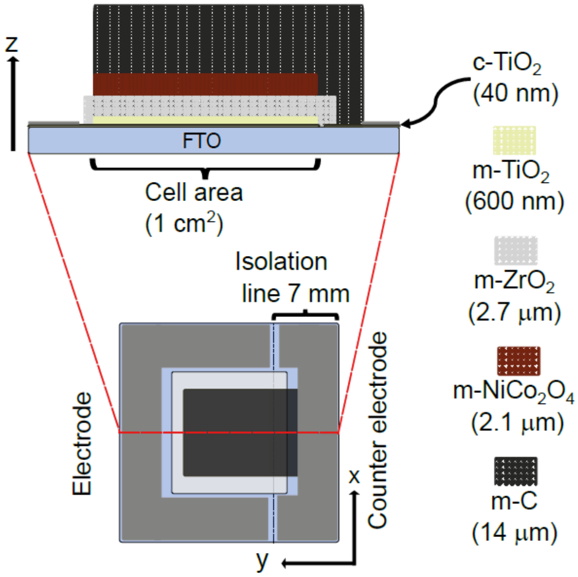

Figure S8. Schematic illustration of the C-PSC configuration.

Table S1. Photovoltaic parameters of the devices with 2.1 μm included in the statistical analysis.

2.1 μm $NiCo_2O_4$	Reverse				Forward			
	PCE (%)	FF (%)	V_{OC} (V)	J_{SC} (mA/cm^2)	PCE (%)	FF (%)	V_{OC} (V)	J_{SC} (mA/cm^2)
1	10.14	49.05	0.939	22.01	5.88	40.13	0.917	15.98
2	11.97	53.32	0.956	23.48	6.81	31.49	0.917	23.56
3	11.13	50.85	0.971	22.53	5.86	30.42	0.947	20.34
4	11.59	51.71	0.952	23.56	7.78	36.09	0.934	23.09
5	11.67	51.91	0.973	23.10	7.43	33.90	0.939	23.36
6	12.38	52.83	0.991	23.65	6.65	34.59	0.920	20.91
7	10.82	46.32	1.003	23.29	6.82	30.85	0.949	23.31
8	10.23	50.24	0.915	22.26	5.66	34.32	0.854	19.31
9	10.25	47.29	0.961	22.55	8.67	41.35	0.917	22.85
10	10.02	44.10	0.961	23.65	6.58	32.10	0.893	22.96
**Mean:**	**11.02**	**49.76**	**0.962**	**23.01**	**6.81**	**34.52**	**0.919**	**21.57**

Table S2. Photovoltaic parameters of the devices with 1.1 μm included in the statistical analysis.

1.1 μm $NiCo_2O_4$	Reverse				Forward			
	PCE (%)	FF (%)	V_{OC} (V)	J_{SC} (mA/cm^2)	PCE (%)	FF (%)	V_{OC} (V)	J_{SC} (mA/cm^2)
1	7.35	39.49	0.873	21.32	6.56	36.18	0.846	21.43
2	7.82	42.65	0.862	21.29	5.99	33.26	0.805	22.36
3	8.85	48.75	0.890	20.41	8.49	44.47	0.880	21.69
4	8.55	49.52	0.873	19.79	7.99	47.52	0.809	20.77
5	9.70	50.93	0.900	21.16	8.52	45.74	0.836	22.26
6	7.57	46.56	0.880	18.48	7.46	45.96	0.817	19.88
7	8.58	49.01	0.906	19.32	7.50	44.20	0.841	20.19
8	7.96	41.87	0.947	20.08	7.54	38.38	0.945	20.80
9	6.99	37.58	0.942	19.74	6.70	33.99	0.976	20.20
10	8.38	44.48	0.912	20.67	7.98	41.37	0.908	21.26
**Mean:**	**8.18**	**45.08**	**0.898**	**20.23**	**7.47**	**41.11**	**0.866**	**21.08**

Table S3. Photovoltaic parameters of triple-stack reference devices included in the statistical analysis.

Reference	Reverse				Forward			
	PCE (%)	FF (%)	V_{OC} (V)	J_{SC} (mA/cm^2)	PCE (%)	FF (%)	V_{OC} (V)	J_{SC} (mA/cm^2)
1	7.77	42.59	0.854	21.36	6.39	35.43	0.800	22.56
2	7.94	41.60	0.900	21.22	5.46	31.17	0.853	20.52
3	8.78	48.29	0.856	21.24	7.62	44.34	0.805	21.35
4	8.50	45.12	0.869	21.67	7.60	38.34	0.873	22.71
5	6.93	43.61	0.841	18.90	7.22	43.79	0.807	20.41
6	7.76	42.65	0.883	20.59	8.01	40.76	0.891	22.06
7	9.01	47.27	0.890	21.42	7.36	39.72	0.863	21.47
8	9.07	47.18	0.854	22.53	7.91	43.56	0.812	22.36
9	6.36	37.60	0.839	20.17	5.44	36.53	0.768	19.38
10	6.50	39.15	0.905	18.35	4.22	28.41	0.843	17.62
**Mean:**	**7.86**	**43.51**	**0.869**	**20.75**	**6.72**	**38.21**	**0.832**	**21.05**

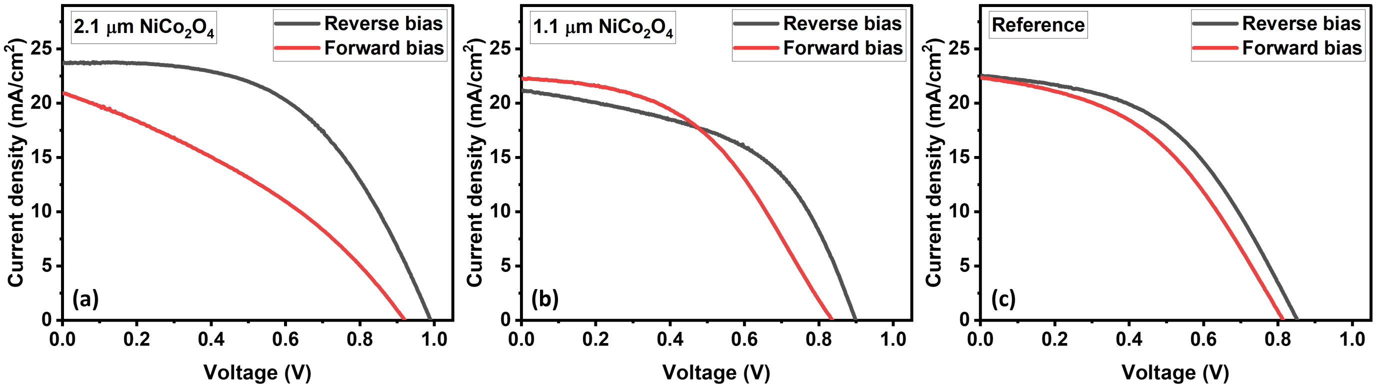

Figure S9. Current – voltage curves recorded at 50 mV s^{-1} for the champion cells for the three configurations, illustrating that the hysteresis increases with the high-capacitance $NiCo_2O_4$ layer thickness.

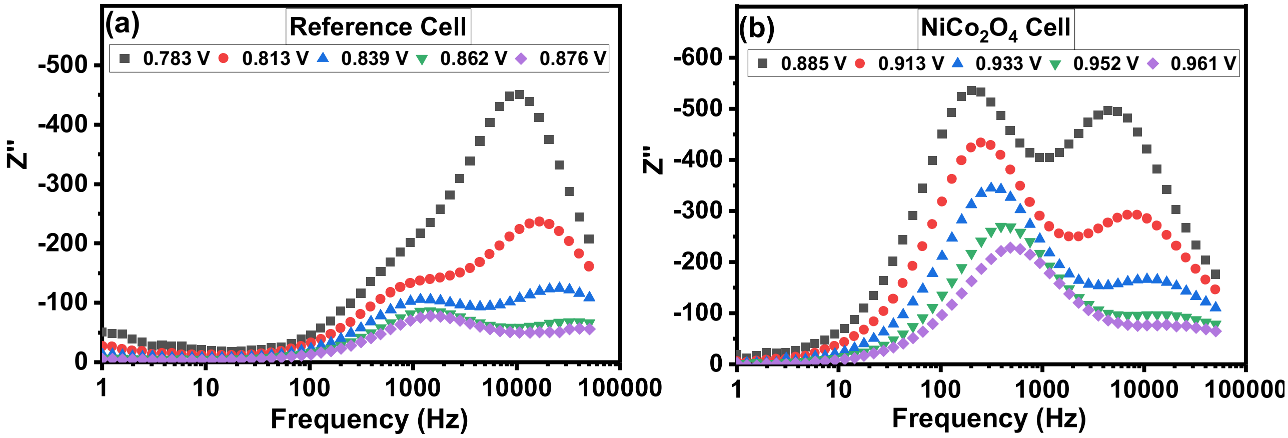

Figure S10. Cole-Cole diagrams from the impedance spectroscopy measurements as a function of V_{OC} for (a) reference cells; and (b) cells with the additional $NiCo_2O_4$ interlayer

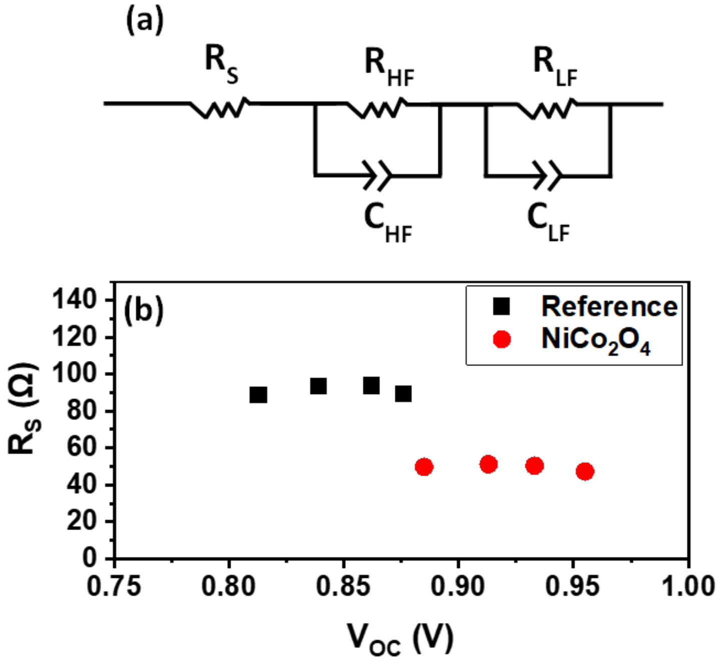

Figure S11. (a) Equivalent circuit model used to fit the impedance spectroscopy data, consisting of a series resistance (R_S) in series with two parallel RC elements. A constant phase element (CPE) is used to account for non-ideal capacitive behavior in the high- and low-frequency regions.
(b) Series resistance (R_S) extracted from impedance spectroscopy under blue illumination, plotted as a function of open-circuit voltage (V_{OC}) for reference cells and $NiCo_2O_4$-integrated cells.

Table S4. Ideality factors determined from the high and low frequency resistance for both solar cell configurations, and as a function of the wavelength of the LED.

Ideality factor	LED	Reference cells	$NiCo_2O_4$
From R_{HF}	White	1.898	1.707
	Blue	1.884	1.561
	Red	1.441	1.361
From R_{LF}	White	2.993	3.196
	Blue	2.724	3.513
	Red	2.909	2.892

References

[1] Kayani, Z. N.; Butt, M. Z.; Riaz, S.; Naseem, S. Synthesis of NiO Nanoparticles by Sol-Gel Technique. Mater. Sci.-Pol. 2018, 36 (4), 547−552. DOI: 10.2478/msp-2018-0080.

[2] Venkatachalam, V.; Alsalme, A.; Alghamdi, A.; Jayavel, R. High Performance Electrochemical Capacitor Based on $MnCo_2O_4$ Nanostructured Electrode. J. Electroanal. Chem. 2015, 756, 94−100. DOI: 10.1016/j.jelechem.2015.08.019.

[3] Venkatachalam, V.; Alsalme, A.; Alghamdi, A.; Jayavel, R. Hexagonal-like $NiCo_2O_4$ Nanostructure Based High-Performance Supercapacitor Electrodes. Ionics 2017, 23 (4), 977–984. DOI: 10.1007/s11581-016-1868-x.

[4] Brault, P.; Ranson, P.; Estrade-Szwarckopf, H.; Rousseau, B. Chemical Physics of Fluorine Plasma-Etched Silicon Surfaces: Study of Surface Contaminations. J. Appl. Phys. 1990, 68 (4), 1702−1709. DOI: 10.1063/1.346625.

[5] Charlier, J.; Detalle, V.; Valin, F.; Bureau, C.; Lécayon, G. Study of Ultrathin Polyamide-6,6 Films on Clean Copper and Platinum. J. Vac. Sci. Technol. A 1997, 15 (2), 353−364. DOI: 10.1116/1.580491.

[6] Bui, L. N.; Thompson, M.; McKeown, N. B.; Romaschin, A. D.; Kalman, P. G. Surface Modification of the Biomedical Polymer Poly(ethylene Terephthalate). Analyst 1993, 118 (5), 463−474. DOI: 10.1039/AN9931800463.

[7] Carter, J.; Schweitzer, G. K.; Carlson, T. A. Experimental Evaluation of a Simple Model for Quantitative Analysis in X-ray Photoelectron Spectroscopy. *J. Electron Spectrosc. Relat. Phenom.* **1974**, *5* (1), 827−835. DOI: 10.1016/0368-2048(74)85055-3.

[8] McIntyre, N. S.; Cook, M. G. X-ray Photoelectron Studies on Some Oxides and Hydroxides of Cobalt, Nickel, and Copper. Anal. Chem. 1975, 47 (13), 2208–2213. DOI: 10.1021/ac60363a034.

[9] Hagelin-Weaver, H. A. E.; Hoflund, G. B.; Minahan, D. M.; Salaita, G. N. Electron Energy Loss Spectroscopic Investigation of Co Metal, CoO, and Co_3O_4 Before and After Ar^+

Bombardment. Appl. Surf. Sci. 2004, 235 (4), 420−448. DOI: 10.1016/j.apsusc.2004.02.062.

[10] Beccaria, A. M.; Poggi, G.; Castello, G. Influence of Passive Film Composition and Sea Water Pressure on Resistance to Localised Corrosion of Some Stainless Steels in Sea Water. Br. Corros. J. 1995, 30 (4), 283−287. DOI: 10.1179/bcj.1995.30.4.283.

[11] Oku, M.; Hirokawa, K. X-ray Photoelectron Spectroscopy of Co_3O_4, Fe_3O_4, Mn_3O_4, and Related Compounds. J. Electron Spectrosc. Relat. Phenom. 1976, 8 (5), 475−481. DOI: 10.1016/0368-2048(76)80034-5.

[12] Wanger, C. D.; Riggs, W. M.; Davis, L. E.; Moulder, J. F.; Muilenberg, G. E. Handbook of X-ray Photoelectron Spectroscopy; Perkin-Elmer Corp.: Eden Prairie, MN, USA, 1979.

[13] Biesinger, M. C.; Payne, B. P.; Lau, L. W. M.; Gerson, A.; Smart, R. S. C. X-ray Photoelectron Spectroscopic Chemical State Quantification of Mixed Nickel Metal, Oxide and Hydroxide Systems. Surf. Interface Anal. 2009, 41 (4), 324−332. DOI: 10.1002/sia.3026.

[14] Manalu, A.; Tarigan, K.; Humaidi, S.; Ginting, M.; Sebayang, K.; Rianna, M.; Hamid, M.; Subhan, A.; Sebayang, P.; Manalu, I. P. Synthesis, Microstructure and Electrical Properties of $NiCo_2O_4$/rGO Composites as Pseudocapacitive Electrode for Supercapacitors. Int. J. Electrochem. Sci. 2022, 17 (3), 22036. DOI: 10.20964/2022.03.11.

[15] Cui, B.; Lin, H.; Liu, Y. Z.; Li, J. B.; Sun, P.; Zhao, X. C.; Liu, C. J. Photophysical and Photocatalytic Properties of Core-Ring Structured $NiCo_2O_4$ Nanoplatelets. J. Phys. Chem. C 2009, 113 (32), 14083–14087. DOI: 10.1021/jp900028t.

[16] Jia, C.; Yang, F.; Zhao, L.; Cheng, G.; Yang, G. Temperature-Dependent Electrical Transport Properties of Individual $NiCo_2O_4$ Nanowire. Nanoscale Res. Lett. 2019, 14 (1), 10. DOI: 10.1186/s11671-018-2844-3.
